# Cohort profile: the Food Chain Plus (FoCus) cohort

**DOI:** 10.1007/s10654-022-00924-y

**Published:** 2022-10-16

**Authors:** Corinna Geisler, Kristina Schlicht, Carina Knappe, Nathalie Rohmann, Katharina Hartmann, Kathrin Türk, Ute Settgast, Dominik M. Schulte, Tobias Demetrowitsch, Julia Jensen-Kroll, Alina Pisarevskaja, Fynn Brix, Bärbel Gruber, Gerald Rimbach, Frank Döring, Philip Rosenstiel, Andre Franke, Stefan Schreiber, Christian H. C. A. Henning, Wolfgang Lieb, Ute Nöthlings, Karin Schwarz, Matthias Laudes

**Affiliations:** 1 Institute of Diabetes and Clinical Metabolic Research, University Medical Center Schleswig-Holstein, Kiel University, Düsternbrooker Weg 17, 24105 Kiel, Germany; 2 Division of Endocrinology, Diabetes and Clinical Nutrition, Department of Internal Medicine I, University Medical Center Schleswig-Holstein, Campus Kiel, Kiel, Germany; 3 Division of Food Technology, Institute of Human Nutrition and Food Science, Kiel University, Kiel, Germany; 4 Division of Food Science, Institute of Human Nutrition and Food Science, Kiel University, Kiel, Germany; 5 Division of Molecular Prevention, Institute of Human Nutrition and Food Science, Kiel University, Kiel, Germany; 6 Institute of Clinical Molecular Biology (IKMB), Kiel University, Kiel, Germany; 7 Division of Agricultural Politics, Institute of Agricultural Economics, Kiel University, Kiel, Germany; 8 Institute of Epidemiology, University Medical Center Schleswig-Holstein, Campus Kiel, Kiel, Germany; 9 Division of Nutrition and Food Sciences, Nutritional Epidemiology, University of Bonn, Bonn, Germany

**Keywords:** Metabolic inflammation, Disease progression, Cohort, Longitudinal

## Abstract

The Food Chain Plus (FoCus) cohort was launched in 2011 for population-based research related to metabolic inflammation. To characterize this novel pathology in a comprehensive manner, data collection included multiple omics layers such as phenomics, microbiomics, metabolomics, genomics, and metagenomics as well as nutrition profiling, taste perception phenotyping and social network analysis. The cohort was set-up to represent a Northern German population of the Kiel region. Two-step recruitment included the randomised enrolment of participants via residents’ registration offices and via the Obesity Outpatient Centre of the University Medical Center Schleswig–Holstein (UKSH). Hence, both a population- and metabolic inflammation- based cohort was created. In total, 1795 individuals were analysed at baseline. Baseline data collection took place between 2011 and 2014, including 63% females and 37% males with an age range of 18–83 years. The median age of all participants was 52.0 years [IQR: 42.5; 63.0 years] and the median baseline BMI in the study population was 27.7 kg/m^2^ [IQR: 23.7; 35.9 kg/m^2^]. In the baseline cohort, 14.1% of participants had type 2 diabetes mellitus, which was more prevalent in the subjects of the metabolic inflammation group (MIG; 31.8%). Follow-up for the assessment of disease progression, as well as the onset of new diseases with changes in subject’s phenotype, diet or lifestyle factors is planned every 5 years. The first follow-up period was finished in 2020 and included 820 subjects.

**Supplementary Information:**

The online version contains supplementary material available at 10.1007/s10654-022-00924-y.

## Background

The Food Chain Plus (FoCus) cohort was launched in 2011 for population-based research in metabolic inflammation. Today’s nutrition, high in energy, fat and simple sugars, interacting sedentary lifestyles and unhealthy environment, is associated with chronic metabolic inflammation, also termed metaflammation. Metabolic inflammation is observed, for example, in some obese subjects without infection, and could lead to severe comorbidities [1]. While low-grade chronic inflammation was initially thought to be the consequence of metabolic disturbances, recent research suggests that inflammatory reactions involving the innate immune system may contribute causally to common metabolic diseases such as obesity, type 2 diabetes and atherosclerosis. Indeed, several pharmaceutical companies are currently developing anti-inflammatory drugs for the treatment of cardiometabolic diseases [2, 3]. Current nutrition and sport sciences are also keenly interested in the development of anti-inflammatory diets and life-style interventions as preventive strategies to counter the growing world-wide prevalence of these diseases. Prospective cohort studies are defined by recruitment of subjects and collection of baseline data before any participants have developed the outcomes of interest. Well-known examples of this strategy are the Framingham Study [4], funded in 1948, a long-term ongoing cardiovascular cohort study of residents of the City of Framingham in the United States, the Swiss HIV Cohort study [5], and the Danish Cohort study of psoriasis and depression [6]. However, to the best of our knowledge, a cohort study specifically addressing metabolic inflammation is missing.

## The Food Chain Plus (FoCus) cohort study: rationale and aims

**Fig. 1:**
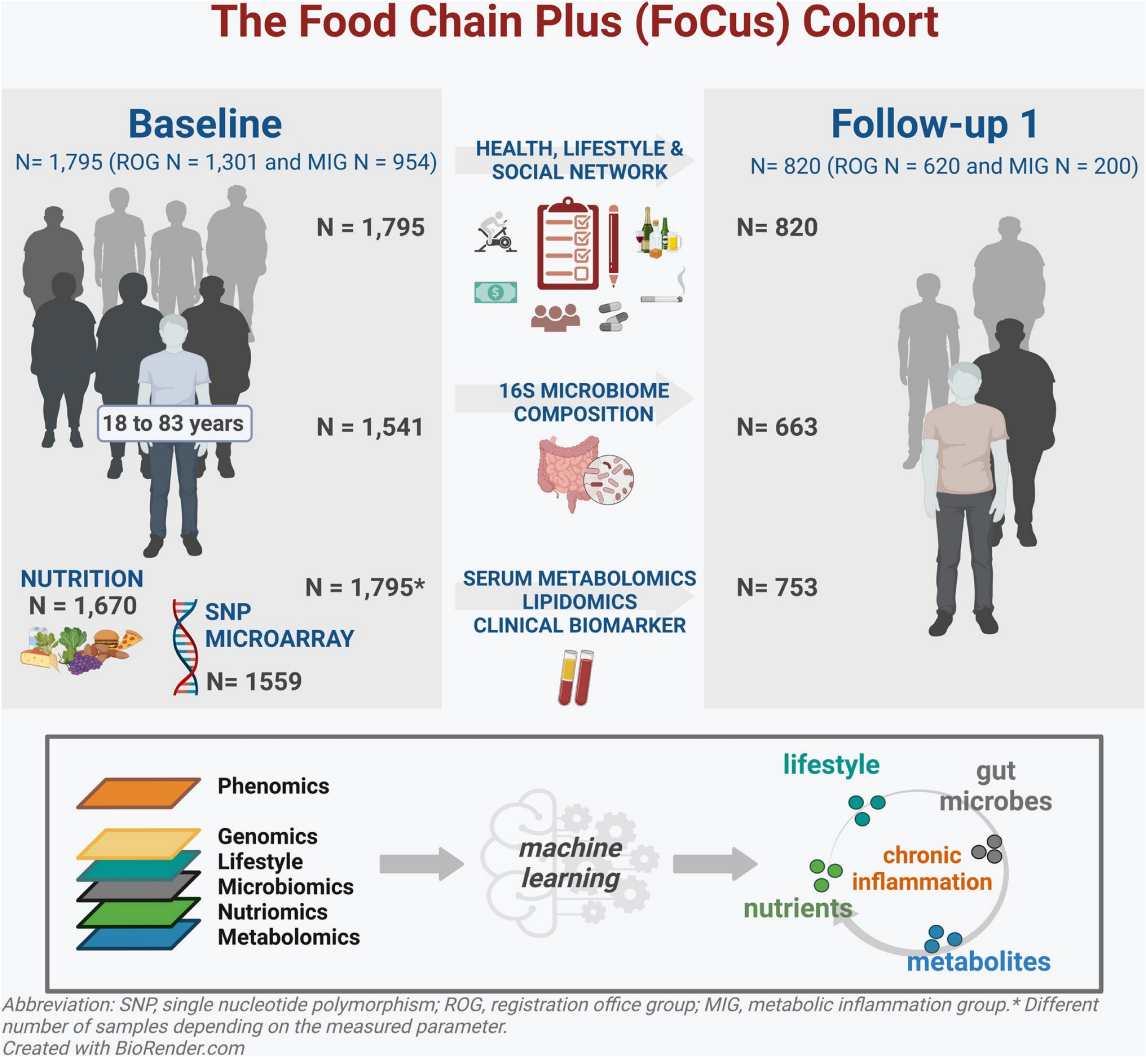
Study design of the FoCus cohort

To characterize the emerging pathology of metaflammation in a comprehensive manner in a Northern Germany Cohort, the Food Chain Plus (FoCus) cohort study was instigated. Data collection included multiple omics modalities including phenomics, metabolomics, genomics and metagenomics (see Fig. 1). The aims of the study are to identify determinants of the development and progression of (A) diseases based on chronic inflammation (e.g. type 2 diabetes mellitus); (B) associated comorbidities (e.g. cardiovascular disease, retinopathy, neuropathy and nephropathy); and (C) developing comorbidities, such as gastrointestinal, musculoskeletal and respiratory diseases. In addition, the reciprocal impacts of these diseases, comorbidities and lifestyle factors on different omics will be defined. Future re-calls are envisaged at 5-year intervals in order to accrue longitudinal data permitting examination of the progression from pre-disease to manifest metabolic and cardiovascular disease, of the course of established diseases and of the development of disease-related complications. The planned follow-up examinations offer a wide range of opportunities to relate progressive changes in disease biomarkers or existing diseases, as well as the onset of new diseases, to changes in subjects’ phenotypes, diets or lifestyle factors, as external environmental factors. In addition, the microbiomic, metabolomic and genotype data will offer further potential to investigate their direct or indirect influence on the development of certain diseases.

## Study design and methodology

### Study population and recruitment

**Fig. 2:**
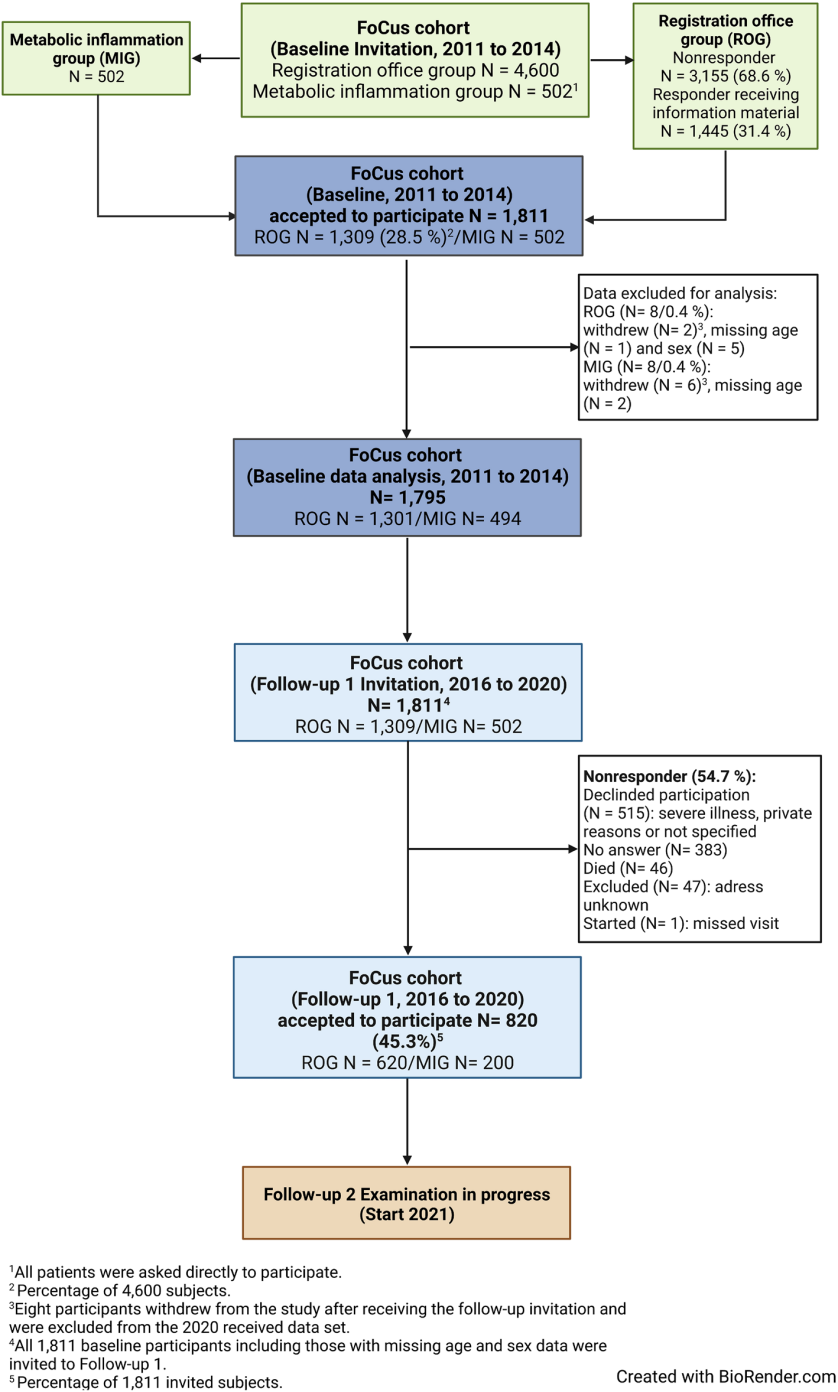
Flow chart of the FoCus cohort

In order to obtain a sufficient cohort to study metabolic inflammation in the future, a two-step process was planned for FoCus cohort recruitment: [A] Seventy-five percent of the subjects were recruited randomly (n = 4,600; assuming a response rate between 30.0 and 40.0%) from the population via the local registration offices in the Kiel area, ensuring a cross-sectional representation of the population in Kiel, the capitol of the federal state Schleswig–Holstein in Northern Germany, and the area within a radius of 15-kms. These subjects are defined as the registration office group (ROG). 28.5% (n = 1309) of the 4600 invited subjects agreed to participate. [B] 25% of FoCus particpants were patients with metabolic inflammation recruited from the Outpatient Centre of the Division of Endocrinology, Diabetes and Clinical Nutrition of the University Medical Center of Schleswig–Holstein in Kiel (UKSH). These patients also lived in the Kiel area. The reason to include these subjects was to ensure a significant number of subjects with clinically manifest metabolic inflammation at baseline, in order to identify biomarkers or pathologies which can be followed longitudinally in subjects drawn from the baseline sample. This group is defined as the metabolic inflammation group (MIG). During their regular clinic visits, 502 patients were personally asked whether they would like to participate in the cohort study and all agreed to participate. Data acquisition and management were performed in close collaboration with the popgen Biobank of the Institute of Epidemiology [7]. The detailed study flowchart is presented in Fig. 2.

### Recruitment phase (2011–2014)

During the first recruitment phase referred to as baseline in the following, data were collected between 2011 and 2014 from 1811 adults (18–83 years). Two hundred of the 1,811 subjects were pheno- and geno-typed twice to validate the analytical methods used, through re-invitation six months after initial recruitment. After internal data pre-processing 1795 of the 1811 subjects were eligible for further data analyses (8 withdrew consent to participate, 8 were excluded for data quality reasons), including 1301 (72.5%) residents (ROG) from the regional registration office and 494 (27.5%) patients (MIG) from the Obesity Outpatient Centre (see Fig. 2).

Before its start, the study was approved by the local ethics committee of the Kiel University (A156-03/Date 2011/07/28) and was registered under the clinical trial number DRKS00005285 at the German Clinical Trials Register in Cologne. All participants were informed about the nature of the study and the study procedure including anthropometric measures, biomaterial sampling, dietary and lifestyle assessment as well as data handling. All participants had appropriate time to consider whether they wanted to take part in the study. Participants were also informed that they could withdraw from the study at any time without giving reasons. Data collected up to that point were then removed from the database accordingly. After that procedure, all participants who provided written informed consent were included into the FoCus study. Participants were also asked for their consent to be contacted in the future for follow-up visits.

### Data collection

Participants within the cohort underwent an internal and nutritional medical phenotyping program (Tables 1, 2 and 3) including, for example, anthropometric measurements, medical history, sociodemographic data, analysis of metabolic and inflammatory markers in blood samples and evaluations of a food frequency questionnaire.

**Table 1:** Physical and medical examinations conducted and collected biomaterial at baseline and first follow-up (Follow-up 1)

Instrument	Time points	
	Baseline (N = 1795)	Follow-up 1 (N = 820)
*Anthropometry*		
Body weight and body height	× (measured)	× (self-reported)
Waist and hip circumference	×	
Body composition analysis (BIA)	× (N = 46)	
Clinical Characteristics		
Blood pressure	×	
Handgrip strength	× (N = 1751)	
*Biomaterial*		
Serum	×	×
EDTA whole blood	×	
EDTA plasma	×	
EDTA cellular components	×	
Lithium heparin plasma	×	
Urine	×	
Native stool	×	×
*Additional examinations*		
Bitter test^a^	× (N = 1778)	
Saline test^a^	× (N = 1,77)	
Magnetic resonance imaging (MRI) of the brain	× (N = 54)	

^a^For details please see section “Additional examinations” and References [28, 29]

**Table 2:** Blood and urine parameters determined at baseline and first follow-up (Follow-up 1)

Parameter	Method	Time points	Follow-up 1 (N = 820)
		Baseline (N = 1795)	
*Lithium–Heparin plasma*			
Glucose	Photometry	× (N = 1789)	
Triglycerides	Enzymatic colour test	× (N = 1790)	
Cholesterol total	Photometry	× (N = 967)	
Low density lipoprotein cholesterol (LDL-c)	Photometry	× (N = 123)	
High density lipoprotein cholesterol (HDL-c)	Photometry	× (N = 123)	
C-reactive protein (CRP)	Immunoturbidimetric test	× (N = 1209)	
Interleukin 6 (IL-6)	Electrochemiluminescence immunoassay (ECLIA)	× (N = 1453)	
Insulin	Electrochemiluminescence immunoassay (ECLIA)	× (N = 1778)	
*Serum*			
Lipoprotein a	Immunoturbidimetric test	× (N = 675)	
Untargeted metabolomics	Fourier transform—Ion Cyclotron resonance—mass spectrometry (FT-ICR-MS)	× (N = 1747)	
Coenzyme Q10	Enzyme-linked Immunosorbent Assay (ELISA)	× (N = 969)	
Thyreoperoxidase (TPO)	Enzyme-linked immunosorbent assay (ELISA)	× (N = 339)	
Antibodies against cyclic citrulline peptides (CCP)	Enzyme-linked immunosorbent assay (ELISA)	× (N = 475)	
Antinuclear antibodies (ANA)	Enzyme-linked immunosorbent assay (ELISA)	× (N = 765)	
wingless-Type MMTV Integration Site Family Member 5A (WNT5A)	Enzyme-linked immunosorbent assay (ELISA)	× (N = 883)	
Secreted frizzled‐related protein 5 (SFRP5)	Enzyme-linked immunosorbent assay (ELISA)	× (N = 836)	
Vitamin D3 (25-OH)	Liquid chromatography-tandem mass spectrometry (LC–MS/MS)	× (N = 385)	
Anti-Saccharomyces cerevisiae antibody	Indirect immunofluorescence test (IIFT)	× (N = 1786)	
Glycoprotein 2 Antibody	Enzyme-linked immunosorbent assay (ELISA)	× (N = 1786)	
Bile acid profile	Liquid chromatography with tandem mass spectrometry (LC–MS/MS)	× (N = 434)	
Nicotineamid (NAM)	High performance liquid chromatography and tandem mass spectrometry (HPLC/CTC-PAL)	× (N = 531)	
Nicotinic acid (NA)	High performance liquid chromatography and tandem mass spectrometry (HPLC/CTC-PAL)	× (N = 531)	
Tryptophan	Liquid chromatography-tandem mass spectrometry (LC–MS/MS)	× (N = 531)	
Tumor necrosis factor α (TNF-α)	Enzyme-linked immunosorbent assay (ELISA)	× (N = 107)	
Fibroblast growth factor 21 (FGF-21)	Enzyme-linked immunosorbent assay (ELISA)	× (N = 246)	
Fetuin-A	Enzyme-linked immunosorbent assay (ELISA)	× (N = 10)	
Myostatin	Enzyme-linked immunosorbent assay (ELISA)	× (N = 10)	
Osteopontin	Enzyme-linked immunosorbent assay (ELISA)	× (N = 10)	
Soluble dipeptidylpeptidase-4 (sDPP-4)	Enzyme-linked immunosorbent assay (ELISA)	× (N = 451)	
*EDTA-plasma*			
Genomics	Infinium Assay Lab setup and procedures guide from illumina	× (N = 1,559)	
*Urine*			
Untargeted metabolomics	Fourier transform—ion cyclotron resonance—mass spectrometry (FT-ICR-MS)	× (N = 891)	
*Stool*			
Gut-microbiome	16S rDNA amplicon sequencing	× (N = 1541)	× (N = 663)

**Table 3:** Questionnaires conducted at baseline and first follow-up (Follow-up 1)

Instrument	Time-points	
	Baseline (N = 1795)	Follow-up 1 (N = 820)
*Questionnaire of medical, sociodemographic and socioeconomic variables*		
Sociodemographic factors	×	×
Medical history (self-reported)	×	×
Family history	×	
Medication intake (regular, last 5 days before examination, which?)	×	×
Antibiotic intake		×
Smoking behaviour	×	×
Employment status	×	
School education	×	
Cancer diseases	×	×
Coronary heart disease	×	×
Vascular diseases	×	×
Diseases of internal organs (lung, gall bladder, intestine and liver)	×	×
Organ transplantation	×	×
Metabolic diseases	×	×
Skin diseases	×	×
Neurological diseases	×	×
Diseases of the auditory apparatus	×	
Allergies	×	×
Dental health	×	×
Women's health (pregnancy, delivery, menstruation and menopause)	×	×
Bone health		×
*Questionnaire on the frequency of consumption of food, activity and sleep*		
Food frequency questionnaire	×	
Daily activity (do it yourself, household, stair climbing)	×	
Sports activity (sports, cycling, gardening, walking)	×	
Sleep (night, day)	×	
Watching TV	×	
Short nutrition assessment		×
*Questionnaire on social networks*		
Social networks	× (N = 677)	

### Examinations

All examinations were performed at the study centre by trained medical staff according to the study-specific standard operating protocol.

#### Anthropometric and blood pressure measurements

Height and weight were measured during the clinical investigation and body mass index (BMI = weight (kg)/ height (m)^2^) was calculated. BMI classes were stratified according to the World Health Organization (WHO) [8] as follows: underweight (< 18.5 kg/m^2^), normal weight (18.5–24.9 kg/m^2^), overweight (25–29.9 kg/m^2^), obesity grade I (30–34.9 kg/m^2^), obesity grade II (35–39.9 kg/m^2^) and obesity grade III (≥ 40 kg/m^2^). Participants were weighed without shoes and wearing light clothes using digital scales (Tanita BC-418 MA, Tanita Europe BV, Amsterdam, Netherlands). The weight was determined to the nearest 0.1 kg. Height was measured to the nearest 1 cm using a stadiometer (seca GmbH&Co.KG, Hamburg, Germany). Waist circumference was measured at the approximate midpoint between the lower margin of the last palpable rib and the top of the iliac crest and hip circumference around the widest portion of the buttocks according to the WHO [9]. All physical examinations were performed by trained medical staff and each measurement was repeated twice by the same staff member. For data analyses, mean values were calculated. Body fat analysis were performed using a single frequency body composition analyser (Tanita BC-418 MA, Tanita Europe BV, Amsterdam, Netherlands; bioimpedance analysis (BIA)) in standing position. The advantages of the BIA analysis technique are its ease of use and the non-invasive mode of measurement.

After a resting period of 5–10 min in sitting position, measurement of systolic and diastolic blood pressure was undertaken twice by sphygmomanometer (weight adopted blood pressure cuffs; BOSCH + SOHN GmbH u. Co. KG, Jungingen, Germany) and stethoscope, with 3-min between measurements. The mean of the two blood pressure measurements was used for further analyses.

#### Handgrip strength determination

Muscle strength was assessed by measuring handgrip strength (HGS) using a MAP 80K1 handgrip dynamometer (Kern & Sohn GmbH, Balingen, Germany). The HGS was measured in a sitting position and the forearm was angled at 90° to the upper arm. Subjects were asked to squeeze the handle of the dynamometer as strongly as they could. Isometric HGS was measured three times for left and right hand, in turn. The participants were also asked about their dominant hand. The mean of the three measurements was calculated for the right and left side. For future analyses the mean of the dominant hand will be used.

### Laboratory analyses

Fasting blood samples were obtained by venepuncture after an overnight fast (average fasting time 10.75 ± 5.3 h) for biochemical analysis of metabolic and inflammatory markers. Midstream urine samples for metabolomics were collected on the day of the visit. Stool samples for microbiome analysis were collected by participants prior to the visit (Table 2).

#### Blood sample analyses

C-reactive protein (CRP), interleukin-6 (IL-6), fasting glucose, fasting insulin and triglycerides were analysed in the central laboratory of the UKSH in Kiel on the day of collection. HOMA-IR was calculated (Homeostasis Model Assessment Insulin Resistance = fasting glucose (mg/dL) × fasting insulin (μU/mL)/405) as an index of insulin sensitivity. Blood samples for central laboratory analysis were stored at 4° C until transport.

All other blood samples and other biomaterials were collected and processed following internal laboratory standardized operating procedures. In general, blood samples were centrifuged, separated and aliquoted. For the analysis of genotypes, whole blood samples were frozen. Aliquoted samples were stored at -80 °C.

Protein levels of wingless-Type MMTV Integration Site Family Member 5A (WNT5A), secreted frizzled‐related protein 5 (sFRP 5), myostatin, fetuin-A, osteopontin and fibroblast growth factor 21 (FGF-21) were assessed in subgroups of participants using the following commercially available ELISA kits: WNT5A (SEP549Hu), sFRP5 (SEC842Hu), myostatin (SEB653Hu), fetuin-A (SEA178Hu), osteopontin (SEA899Hu) and FGF-21 (SEC918Hu) all from the Cloud-Clone Corp. (Wuhan, Peoples Republic of China). The measurements were performed according to the manufacturer’s instructions. Nicotinic acid and Nicotinamide serum levels were measured by liquid chromatography and tandem mass spectrometry (Agilent 1100 HPLC/CTC-PAL Autosampler/Sciex API 4000 Triple Quadrupole) by an external specialized laboratory (Medizinisches Labor Bremen, Bremen, Germany) (for methods see Table 2).

#### Gut microbiome analyses

Initially, stool samples of all subjects submitted to the study centre between 2011 and 2015 were stored at – 80 °C until further analysis. The subsequent preparation and sequencing of the samples was carried out by the Institute for Clinical Molecular Biology (IKMB) at the Kiel University as described by Heinsen et al. [10].

#### DNA extraction from stool samples

DNA was extracted from stool samples using the QIAamp DNA Stool Mini Kit and the QIAcube system (both from Qiagen). After thawing, about 200 mg of sample material was used and transferred to bead-beating tubes (Garnet, 0.7 mm) which were filled with 1.1 ml of ASL lysis buffer. This solution in the tubes was homogenized in SpeedMill PLUS (Analytik Jena) for 45 s at 50 Hz. The samples were then heated to 95 °C. for 5 min. All further steps were continued according to the manufacturer's protocol.

#### Amplicon sequencing of bacterial 16S rRNA and quality control

During preparation for sequencing, the variable V1-V2 region of the 16S rRNA gene was amplified by polymerase chain reaction (PCR), using a pair of primers 27F/338R with an individual combination of two barcodes according to the dual barcoding approach of Caporaso et al. [11]. The SequalPrep Normalization Plate Kit (Thermo Fisher Scientific) according to the manufacturer's instructions was used to normalize the DNA concentration from the PCR products. This was followed by sequencing of the prepared DNA solution with the Illumina MiSeq device. For this purpose, the individual samples were mixed equimolarly (“pooled”). If there was no error, the generated sequences were assigned to the corresponding samples. Complementary sequences were read in the forward and reverse directions and were combined by the program FLASH. The Illumina company also provides a Q-score for the Miseq device, which is used for quality control. Sequences with a Q-Score below 30 in more than 5% of the nucleotides were sorted out using additional software (UCHIME). After quality control, 1541 samples remained for statistical analyses.

#### Genetic analyses

The laboratory procedures and quality controls were conducted at the Institute of Clinical Molecular Biology (IKMB), Kiel University. Genotyping was performed according to the Infinium Assay Lab Setup and Procedures Guide from illumina (December 2017), using the Infinium iScan OmniExpressExome BeadChip array, build 37/hg19 (illumina, San diegi, CA, USA). The BeadChips were scanned and imaged at two wavelengths using the iScan two-channel microarray scanner. The image files generated were further processed by the iScan Control Software and GenomeStudio software and randomly assembled beadtypes were decoded and their corresponding SNPs were identified [12].

#### Quality controls

Quality control was conducted using R version 3.1.0 beta and PLINK (whole genome association analysis toolset) version 1.07 [13] and version 1.90 beta [14]. 942,280 single nucleotide polymorphisms (SNPs) in 1,713 samples were collected. All individuals had a genotype call rate of > 95% over the cohort. For each sample pair, identity by descent (IBD) was calculated. Here, a threshold of IBD > 0.185 was used to exclude strong relatedness among individuals, which could otherwise lead to biased estimations in the association analysis. For the calculation of IBD the SNPRelate–package [15] of R was used and a maximum likelihood approach was applied.

SNPs with missingness > 5% over the cohort were excluded from the data set. This was the case for 2591 SNPs. Hardy–Weinberg Equilibrium (HWE) was used to identify SNPs with genotyping errors and SNPs, of which the observed cohort’s allele frequency did significantly deviate from the expected with a *p*-value > 1e−^05^ were discarded. This was the case for 2048 SNPs. After quality control, 937,641 SNPs were left. A minor allele frequency (MAF) threshold was set at 0.05, meaning that only SNPs which occurred in at least 5% of the cohort samples were used for further analyses. This was the case for 601,203 SNPs.

Population outliers were identified based on ethnicity by principal component analysis (PCA) of data merged to Hapmap Phase III data [16] from four different populations (European, East Asian (Beijing, China), East Asian (Tokyo, Japan) and African). All individuals not located in the rectangle of the cohort were defined as population outliers and excluded from analysis. After the quality control, 1559 samples remained for the genetic analyses.

#### Metabolomic analyses

Blood and urine samples were analysed by different analytical approaches. Urine samples were diluted 1:4 with water containing 0.1% formic acid (v/v) and analysed by a quadrupole time of flight mass spectrometer (Bruker, Bremen, Germany) [17]. To enhance reproducibility, data were filtered for metabolites present in at least 10% of all samples with a minimum intensity of 106 counts. For urine metabolomics, 891 volunteer samples were analysed and 140 recall samples (collected six months after the individual’s first recruiting day).

Blood samples were extracted by a modified SIMPLEX approach in accordance to Matyash et al. [18]. Samples were subjected to ultra-high-resolution Fourier Transform—Ion Cyclotron Resonance—mass spectrometry (FT-ICR-MS (Bruker, Bremen, Germany). This approach provides the highest mass accuracy, highest resolution and excellent sensitivity for metabolomics data [19]. For the semi-targeted evaluation, a local database built using different web databases (e.g. KEGG, HMDB) [20, 21] and various original research papers was used. Chemical formulae were assigned based on a mass error < 2 ppm, isotopic fine structure and the seven golden rules. Data were filtered for metabolites that were present in at least 10% of all samples with a minimum intensity of 1 million counts. In total, samples of 1747 subjects (plus 189 recall samples, obtained six months after initial recruitment) were analysed. 2389 different hydrophilic and 1754 lipophilic metabolites in blood were found with all metabolites present in at least 10% of all subjects.

### Questionnaires

Two different questionnaires were used for the assessment of information regarding the medical history, general health, medication intake, nutrition, lifestyle (e.g. activity, sleep, smoking habits), sociodemographic and socioeconomic factors (see Table 3).

#### Questionnaire of medical, sociodemographic and socioeconomic variables

The main FoCus questionnaire (version 1.1 from July 18, 2011) for assessment of medical status was used to retrieve participants’ medical history. The questionnaire was divided into sections focusing on diverse medical conditions. For example, regarding diabetes status, a question asked whether there was diabetes mellitus diagnosed by the general practitioner. This question could be answered with “yes” or “no”. Sub-questions then asked for the type of diabetes. All participants were asked to bring their regular medication to the study visit in the form of a physician's listing or original packaging (including prescribed and over the counter medication). Participants were also asked if they had taken any medication during the past 5 days before the study visit in addition to their regular medication. Beside medical variables, a second part of the FoCus questionnaire was used to record sociodemographic (e.g. school graduation, type of household, children), socioeconomic (e.g. employment situation) and lifestyle (e.g. content of life, smoking) aspects. Data from 1795 subjects were available.

#### Questionnaire on the frequency of consumption of food, activity and sleep

In cooperation with the German Institute for Nutritional Research Potsdam-Rehbrücke (DIFE), a questionnaire was developed to record the nutritional behaviour and nutrient intake of all macro- and micronutrients and nutritional supplements. The European Prospective Investigation into Cancer and Nutrition (EPIC) FFQ according to Schulz et al. [22] was applied as a Food Frequency Questionnaire (FFQ). This retrospective survey method was used to determine eating behaviour over the past 12 months. Structurally, the FFQ was divided into classical food groups. To get a semi-quantitative record of food intake, questions about both frequency of consumption and portion sizes were asked, with questions accompanied by pictures of portions and sizes. In 10% of the subjects, a phone-based 24 h-recall was used to validate the EPIC-12-month FFQ. Nutrition data were calculated as energy density (main macronutrients e.g. protein, fat, carbohydrates and alcohol) or were energy-adjusted by the residual method of Willet et al. [23] to adjust single nutrients to the energy intake of the group. Physical activity (walking, cycling, sports, gardening, do it yourself, cleaning, watch TV and climbing stairs) and sleep were part of the EPIC FFQ [24–26]. The physical activity questionnaire is a short version of a more extensive questionnaire which was tested in one of the Dutch EPIC centres [27]. Nutrition, activity and sleep data from 1670 subjects were available.

### Additional examinations

#### Testing procedure for taste

To assess the taste sensitivity of the probands, whole-mouth tests were performed. Each subject was tested separately. First of all, probands were asked to neutralize their mouths with drinking water. Next, they were provided with 0.02 L tasting solution. This solution contained the synthetic bitter compound 6-n-Propylthiouracil (PROP) in a concentration of 273 mg/500 mL drinking water. After a few seconds of keeping the PROP solution in the oral cavity, the probands marked the perceived intensity on a general labelled magnitude scale (gLMS). This procedure was repeated for the second taste solution, containing the tasting agent sodium chloride (NaCl) in a concentration of 29 g/500 mL drinking water. A labelled magnitude scale (LMS) consists of five tick marks, labelled from bottom to top as ‘barely detectable’, ‘weak’, ‘moderate’, ‘strong’, ‘very strong’, and ‘strongest imaginable’, dividing the scale (0–234 mm) in a quasi-logarithmic manner [28]. However, the LMS can be further modified using a more unspecific top-label ‘strongest imaginable sensation of any kind’ to achieve a more generalized version of it (gLMS), aiming at the avoidance of ceiling effects by the type of sense under study [29]. After probands marked their perceived taste sensation per test solution on the gLMS, the distances were measured in millimetres. Visual inspection of raw data showed strong clusters, therefore probands were stratified into 3 taste sensitivity groups, ‘low’, ‘medium’ and ‘high’, according to their marked taste perception for each drinking solution. For the bitter taste test, cut-offs were set at ‘low < 61 mm’, ‘medium >  = 61 mm and < 107 mm’ and ‘high >  = 107 mm’ and for the salt taste test, cut-offs were set at ‘low < 107 mm’, ‘medium >  = 107 mm and < 151 mm’ and ‘high > 151 mm’. Taste sensitivity data were available for 1789 and 1788 study participants for bitter and salty respectively.

#### Magnetic resonance imaging (MRI) and spectroscopy of the hypothalamus

The MRI examinations were performed on a 3 T MRI scanner (Achieva; Phillips Medical Systems, Eindhoven, the Netherlands) with a 32-channel head coil. T2-weighted fluid-attenuated inversion recovery (FLAIR) images were used to analyse regions of interest (ROIs). Those were in particular the putamen (PUT), the medio basal hypothalamus (MBH) and the amygdala (AMY). The AMY-data was used to normalize PUT- and MBH-data in order to rule out intraindividual differences and to obtain signal ratios. For placement of the MBH-ROIs in coronal T2-weighted FLAIR images, anatomic landmarks such as the third ventricle and the optic tract were used. A voxel size of 0.9 × 1.13 × 3 mm^3^ (echo time [TE] = 160 ms; repetition time [TR] = 12,000 ms) was used. The volumes of the ROIs were drawn manually. For the MBH, amounts covered were circa 2–4 mm^2^, with a circumference of circa 6–7 mm, diameter of circa 3 mm, and thickness of 3 mm.

Spectroscopy data were analysed using the Philips SpectroView package. Multivoxel proton spectroscopy was performed using multiply optimized insensitive suppression train (MOIST) water suppression with voxel size 10 × 10 × 10 mm^3^ (chemical shift imaging point resolved spectroscopy, echo time = 35 ms, repetition time = 2000 ms) and additional sagittal and axial T2-weighted sequences for the MRS planning. The voxels were placed through the bottom of the third ventricle, unilaterally, to obtain separated results. The voxels (l/r) that included most of the MBH were chosen for spectrum analysis. As a marker of neuronal and axonal viability and intensity N-acetyl-aspartate (NAA) was normalized to creatine (Cr) as internal reference. MRI data are available from 54 subjects.

#### Social network analyses

Nutrition and activity behaviour data as well as relevant socio-economic characteristics of probands were used for detailed network analysis. Socio-economic variables in network analyses included age, biological sex, education, household size, and household income. Behavioural and lifestyle data were collected for a person connected to the study participant (= EGO), designated ALTER. ALTER may influence the behaviour of EGO. Thus, information on frequency of dieting (DIET), attitude towards food (AT), nutritional knowledge (KNOW), and frequency of physical activities (SPORT) were collected for the actor whose network and behaviour choices are being modelled (EGO) as well as for all of EGO’s social network contacts (ALTER). In a special social network survey EGO-centric network data were collected from each proband during the study centre visit using a specific computer-based social network questionnaire. The state of the art name generator concept was used to collect the social network data [30]. Three name generator questions were asked: (G1): With whom do you regularly discuss personal problems? (G2): To whom can you turn for help if you have a problem? (G3): With whom do you regularly discuss health-related (especially weight related) problems? For all ALTER mentioned by EGO in response, EGO was asked for their biological sex, age, education, and profession. In a next step EGO was asked to characterize ALTER by (A) ALTER-BMI measured in five categories (1–5) ranging from very slim to very fat; (B) Nutrition knowledge (ALTER-KNOW): 1 = very low, 2 = low, 3 = average, 4 = good, 5 = excellent; (C) Nutritional attitude (ALTER-AT): 1 = food is mainly convenience, 2 = diet has to balance health and convenience aspects, 3 = diet has to be mainly healthy; (D) Frequency of sport activities (ALTER-SPORT) longer than 30 min: 1 = never; 2 = 1–2 per month, 3 = 1 per week, 4 = several times per week; 5 = every day; (E) Diet behaviour (ALTER-DIET): it is asked how often ALTER has adopted a specific diet to lose weight: 1 = never, 2 = 1 time, 3 = 2–3 times; 4 = 4–5 times, 5 =  > 5 times. EGO was also asked about the strength, length, and importance of the relation with ALTER. EGO was also asked to describe the pairwise relations of the ten most important individuals mentioned on a 3 point scale with 0 = do not know each other, 1 = know each other, 2 = know each other very well [30]. Data were calculated as a different network multiplier (NET-Z). NET-Z measured the field strength of different health-relevant behaviours and attitudes (Z = KNOW, DIET, BMI, AT, SPORT) which are prevalent in EGO’s social network and operating on EGO [31]. A possible bias of the method could be that EGO wouldn´t be able to exactly answer the questions regarding dietary behaviour, nutritional knowledge of a friend. Therefore, the answer could be influenced by EGOs own perception and attitudes.

#### Follow-up 1 (2016–2020)

For follow-up 1, the data collection changed from invited visits at the study centre to visits at the general practitioner. The 1811 baseline cohort subjects were invited to participate in follow-up 1 and 45.3% (n = 820; ROG = 620 and MIG = 200) agreed, whereas 54.7% (n = 991) declined to participate in follow-up 1 (see Fig. 2 for details). The popgen biobank attempted to locate persons who had moved by making inquiries at the residents' registration offices. The first 5-year follow-up was completed in 2020.

### Data collection

Participants in the baseline cohort were asked if they were willing to participate in follow-up after 5 years (for more details please see Fig. 2). Both the former participant and his general practitioner received an information letter from the popgen biobank with information regarding the follow-up. With this letter, participants at the same time received a stool (for native stool sample) and blood collection kit (2 times serum) with the corresponding SOPs for the collection procedure while the general practitioner received only an identical blood collection kit (2 times serum). The collected biomaterials were labelled with an individual barcode and returned to the biobank according to the instructions in the collection cover letter. The blood samples were delivered to the study centre within 2 days (Table 1). Participants were also asked to complete the medical questionnaire, which was then returned by post. In this questionnaire the participants should state their height and also weight and they were asked questions regarding their health status, medication intake and nutrition (Table 3). All participants were informed about the nature of the study and the study procedure including biomaterial sampling and data handling. All participants had the appropriate time to consider whether they wanted to take part in the study after being informed. Participants were also informed that if they had any questions, they could ask the principal investigator at any time during an interview about the nature of the study and then decide whether they wanted to participate in the study. Participants were also informed that they could withdrew from the study at any time without giving reasons. After that procedure, all participants who provided written informed consent were included into the follow-up 1 part of the FoCus study. Participants were also asked for their consent to be contacted in the future for further follow-up visits. The follow-up was an addition to the first approval of the local ethics committee of the Kiel University (A156-03/Date 2011/07/28).

## Baseline characteristics of the study population

### Metabolic health

The final FoCus cohort includes 1795 participants with comprehensive data (63.0% female and 37.0% male) for further data analysis. The median age of all participants was 52.0 years (Table 4) with an age range from 18 to 83 years, however MIG subjects were significantly younger than ROG subjects (Table 4). MIG subjects in comparison to ROG subjects were morbidly obese. ROG subjects showed lower waist- and hip-circumferences and biomarker-based indices of metabolic health (e.g. triglycerides, glucose, insulin or HOMA-IR) and inflammation (CRP and IL-6). The observed differences persisted after stratifying the MIG and ROG subjects by sex. Cholesterol and lipoprotein a were not significantly different between MIG and ROG females (see Table S1).

**Table 4:** Demographic, clinical and laboratory characteristics of the FoCus cohort subjects at baseline stratified by type of recruitment

Characteristics	N	Overall, N = 1795^a^	MIG, N = 494^a^	ROG, N = 1301^a^	*p*-Value^b^
Sex	1795/1795				< 0.001
Females		1131.0/1795.0 (63.0%)	370.0/494.0 (74.9%)	761.0/1301.0 (58.5%)	
Males		664.0/1795.0 (37.0%)	124.0/494.0 (25.1%)	540.0/1301.0 (41.5%)	
Missing		0	0	0	
Age (years)	1795/1795	52.0 (42.5, 63.0)	48.0 (40.0, 57.0)	54.0 (44.0, 65.0)	< 0.001
Missing		0	0	0	
Height (cm)	1795/1795	172.0 (166.0, 179.0)	170.0 (164.6, 177.9)	172.0 (167.0, 180.0)	< 0.001
Missing		0	0	0	
Weight (kg)	1795/1795	84.7 (69.8, 105.5)	123.8 (103.3, 144.2)	76.3 (65.8, 88.7)	< 0.001
Missing		0	0	0	
BMI (kg/m^2^)	1795/1795	27.7 (23.7, 35.9)	42.8 (36.7, 48.8)	25.4 (22.7, 28.6)	< 0.001
Range	1795/1795	14.5, 83.2	19.0, 72.7	14.5, 83.2	
Missing		0	0	0	
BMI class	1795/1795				< 0.001
UW (< 18.5 kg/m^2^)		24.0/1795.0 (1.3%)	0.0/494.0 (0.0%)	24.0/1301.0 (1.8%)	
NW (18.5–24.9 kg/m^2^)		570.0/1795.0 (31.8%)	5.0/494.0 (1.0%)	565.0/1301.0 (43.4%)	
OW (25.0–29.9 kg/m^2^)		483.0/1795.0 (26.9%)	25.0/494.0 (5.1%)	458.0/1301.0 (35.2%)	
OBI (30–34.9 kg/m^2^)		240.0/1795.0 (13.4%)	72.0/494.0 (14.6%)	168.0/1301.0 (12.9%)	
OBII (35.0–39.9 kg/m^2^)		140.0/1795.0 (7.8%)	83.0/494.0 (16.8%)	57.0/1301.0 (4.4%)	
OBIII (≥ 40.0 kg/m^2^)		338.0/1795.0 (18.8%)	309.0/494.0 (62.6%)	29.0/1301.0 (2.2%)	
Missing		0	0	0	
Hip-circumference (cm)	1639/1795	108.0 (101.0, 118.0)	129.0 (120.0, 139.0)	105.0 (100.0, 111.2)	< 0.001
Missing		156	142	14	
Waist-circumference (cm)	1660/1795	96.0 (84.0, 112.0)	123.0 (112.0, 134.0)	92.0 (81.0, 103.0)	< 0.001
Missing		135	124	11	
BP systolic (mmHg)	1795/1795	129.1 (12.0)	133.7 (10.9)	127.3 (12.0)	< 0.001
Missing		0	0	0	
BP diastolic (mmHg)	1795/1795	80.4 (6.7)	83.0 (6.7)	79.4 (6.4)	< 0.001
Missing		0	0	0	
Triglycerides (mg/dL)	1790/1795	108.0 (76.0, 152.8)	140.0 (102.5, 194.5)	98.0 (70.0, 139.0)	< 0.001
Missing		5	3	2	
Cholesterol total (mmol/L)	967/1795	4.6 (4.0, 5.2)	4.5 (4.0, 5.1)	4.6 (4.0, 5.2)	0.30
Missing		828	135	693	
LDL-cholesterol (mmol/L)	123/1795	3.1 (2.6, 3.7)	NA (NA, NA)	3.1 (2.6, 3.7)	
Missing		1672	494	1178	
HDL-cholesterol (mmol/L)	123/1795	1.5 (1.3, 1.9)	NA (NA, NA)	1.5 (1.3, 1.9)	
Missing		1672	494	1178	
Lipoprotein a (mg/L)	675/1795	253.0 (145.0, 485.5)	264.0 (154.5, 492.0)	249.5 (136.0, 485.0)	0.17
Missing		1120	263	857	
Glucose (mg/dL)	1789/1795	95.0 (88.0, 104.0)	101.0 (91.0, 117.0)	93.0 (88.0, 101.0)	< 0.001
Missing		6	3	3	
Insulin (mU/L)	1778/1795	10.1 (6.7, 17.8)	19.2 (11.8, 34.0)	8.6 (6.0, 12.9)	< 0.001
Missing		17	7	10	
HOMA-IR	1785/1795	2.4 (1.5, 4.4)	4.8 (2.7, 9.4)	2.0 (1.3, 3.1)	< 0.001
Missing		10	5	5	
CRP (mg/L)	1209/1795	3.2 (1.6, 6.9)	6.4 (3.4, 11.1)	2.3 (1.4, 3.9)	< 0.001
Missing		586	31	555	
IL-6 (pg/mL)	1453/1795	3.7 (2.7, 5.5)	4.9 (3.5, 6.8)	3.4 (2.4, 4.8)	< 0.001
Missing		342	37	305	
Smoking habits	1744/1795				0.030
Never smoking		627.0/1744.0 (36.0%)	143.0/470.0 (30.4%)	484.0/1274.0 (38.0%)	
Previous smoking		673.0/1744.0 (38.6%)	194.0/470.0 (41.3%)	479.0/1274.0 (37.6%)	
Less than 3 months		124.0/1744.0 (7.1%)	35.0/470.0 (7.4%)	89.0/1274.0 (7.0%)	
Smoking		320.0/1744.0 (18.3%)	98.0/470.0 (20.9%)	222.0/1274.0 (17.4%)	
Missing		51	24	27	
School education	1784/1795				< 0.001
University qualification		543.0/1784.0 (30.4%)	84.0/487.0 (17.2%)	459.0/1297.0 (35.4%)	
Technical college qualification		196.0/1784.0 (11.0%)	42.0/487.0 (8.6%)	154.0/1297.0 (11.9%)	
Middle school		613.0/1784.0 (34.4%)	184.0/487.0 (37.8%)	429.0/1297.0 (33.1%)	
Secondary school		417.0/1784.0 (23.4%)	167.0/487.0 (34.3%)	250.0/1297.0 (19.3%)	
No degree		15.0/1784.0 (0.8%)	10.0/487.0 (2.1%)	5.0/1297.0 (0.4%)	
Missing		11	7	4	
Employment	1783/1795				< 0.001
Full time		603.0/1783.0 (33.8%)	147.0/491.0 (29.9%)	456.0/1292.0 (35.3%)	
Part time		354.0/1783.0 (19.9%)	86.0/491.0 (17.5%)	268.0/1292.0 (20.7%)	
Unemployed		108.0/1783.0 (6.1%)	68.0/491.0 (13.8%)	40.0/1292.0 (3.1%)	
Retired		524.0/1783.0 (29.4%)	122.0/491.0 (24.8%)	402.0/1292.0 (31.1%)	
Other		194.0/1783.0 (10.9%)	68.0/491.0 (13.8%)	126.0/1292.0 (9.8%)	
Missing		12	3	9	
Content of life	1764/1795				< 0.001
Very content		403.0/1764.0 (22.8%)	39.0/479.0 (8.1%)	364.0/1285.0 (28.3%)	
Content		959.0/1764.0 (54.4%)	193.0/479.0 (40.3%)	766.0/1285.0 (59.6%)	
Not so content		321.0/1764.0 (18.2%)	185.0/479.0 (38.6%)	136.0/1285.0 (10.6%)	
Not at all content		81.0/1764.0 (4.6%)	62.0/479.0 (12.9%)	19.0/1285.0 (1.5%)	
Missing		31	15	16	
Household type	1780/1795				< 0.001
Living with partner		1314.0/1780.0 (73.8%)	318.0/492.0 (64.6%)	996.0/1288.0 (77.3%)	
Living alone		356.0/1780.0 (20.0%)	130.0/492.0 (26.4%)	226.0/1288.0 (17.5%)	
Other types		110.0/1780.0 (6.2%)	44.0/492.0 (8.9%)	66.0/1288.0 (5.1%)	
Missing		15	2	13	
Children	1785/1795				0.001
No		550.0/1785.0 (30.8%)	181.0/489.0 (37.0%)	369.0/1296.0 (28.5%)	
Yes		1233.0/1785.0 (69.1%)	308.0/489.0 (63.0%)	925.0/1296.0 (71.4%)	
Unknown		2.0/1785.0 (0.1%)	0.0/489.0 (0.0%)	2.0/1296.0 (0.2%)	
Missing		10	5	5	
Diabetes^c^	1791/1795				< 0.001
Normal		1086.0/1791.0 (60.6%)	197.0/493.0 (40.0%)	889.0/1298.0 (68.5%)	
Prediabetes^d^		419.0/1791.0 (23.4%)	124.0/493.0 (25.2%)	295.0/1298.0 (22.7%)	
T1DM		8.0/1791.0 (0.4%)	2.0/493.0 (0.4%)	6.0/1298.0 (0.5%)	
T2DM^e^		253.0/1791.0 (14.1%)	157.0/493.0 (31.8%)	96.0/1298.0 (7.4%)	
Other		25.0/1791.0 (1.4%)	13.0/493.0 (2.6%)	12.0/1298.0 (0.9%)	
Missing		4	1	3	
Hypertension^c^	1774/1795				< 0.001
No		1023.0/1774.0 (57.7%)	159.0/485.0 (32.8%)	864.0/1289.0 (67.0%)	
Yes		751.0/1774.0 (42.3%)	326.0/485.0 (67.2%)	425.0/1289.0 (33.0%)	
Missing		21	9	12	
Dyslipidemia^c^	1755/1795				0.003
No		1234.0/1755.0 (70.3%)	312.0/480.0 (65.0%)	922.0/1275.0 (72.3%)	
Yes		521.0/1755.0 (29.7%)	168.0/480.0 (35.0%)	353.0/1275.0 (27.7%)	
Missing		40	14	26	
Myocardial infarction^c^	1781/1795				0.70
No		1727.0/1781.0 (97.0%)	471.0/487.0 (96.7%)	1256.0/1294.0 (97.1%)	
Yes		54.0/1781.0 (3.0%)	16.0/487.0 (3.3%)	38.0/1294.0 (2.9%)	
Missing		14	7	7	
Cardiac failure^c^	1761/1795				< 0.001
No		1701.0/1761.0 (96.6%)	450.0/481.0 (93.6%)	1251.0/1280.0 (97.7%)	
Yes		60.0/1761.0 (3.4%)	31.0/481.0 (6.4%)	29.0/1280.0 (2.3%)	
Missing		34	13	21	
Liver disease^c^	1773/1795				< 0.001
No		1664.0/1773.0 (93.9%)	434.0/482.0 (90.0%)	1230.0/1291.0 (95.3%)	
Yes		109.0/1773.0 (6.1%)	48.0/482.0 (10.0%)	61.0/1291.0 (4.7%)	
Missing		22	12	10	
Neurological disease^c^	1744/1795				< 0.001
No		1508.0/1744.0 (86.5%)	377.0/479.0 (78.7%)	1131.0/1265.0 (89.4%)	
Yes		236.0/1744.0 (13.5%)	102.0/479.0 (21.3%)	134.0/1265.0 (10.6%)	
Missing		51	15	36	
Stroke^c^	1782/1795				0.74
No		1750.0/1782.0 (98.2%)	484.0/492.0 (98.4%)	1266.0/1290.0 (98.1%)	
Yes		32.0/1782.0 (1.8%)	8.0/492.0 (1.6%)	24.0/1290.0 (1.9%)	
Missing		13	2	11	
Respiratory disease^c^	390/1795				0.007
Asthma		139.0/390.0 (35.6%)	64.0/181.0 (35.4%)	75.0/209.0 (35.9%)	
Chronic bronchitis		130.0/390.0 (33.3%)	73.0/181.0 (40.3%)	57.0/209.0 (27.3%)	
Others		121.0/390.0 (31.0%)	44.0/181.0 (24.3%)	77.0/209.0 (36.8%)	
Missing		1405	313	1092	
Allergic asthma^c^	1769/1795				< 0.001
No		1625.0/1769.0 (91.9%)	420.0/485.0 (86.6%)	1205.0/1284.0 (93.8%)	
Yes		144.0/1769.0 (8.1%)	65.0/485.0 (13.4%)	79.0/1284.0 (6.2%)	
Missing		26	9	17	
Allergic rhinitis^c^	1774/1795				0.32
No		1424.0/1774.0 (80.3%)	385.0/489.0 (78.7%)	1039.0/1285.0 (80.9%)	
Yes		350.0/1774.0 (19.7%)	104.0/489.0 (21.3%)	246.0/1285.0 (19.1%)	
Missing		21	5	16	
Skin disease^c^	436/1795				0.63
Acne		59.0/436.0 (13.5%)	21.0/118.0 (17.8%)	38.0/318.0 (11.9%)	
Atopic eczema		112.0/436.0 (25.7%)	28.0/118.0 (23.7%)	84.0/318.0 (26.4%)	
Light allergy		13.0/436.0 (3.0%)	3.0/118.0 (2.5%)	10.0/318.0 (3.1%)	
Psoriasis		118.0/436.0 (27.1%)	32.0/118.0 (27.1%)	86.0/318.0 (27.0%)	
Others		134.0/436.0 (30.7%)	34.0/118.0 (28.8%)	100.0/318.0 (31.4%)	
Missing		1359	376	983	
IBD^c^	1773/1795				0.092
No		1670.0/1773.0 (94.2%)	468.0/489.0 (95.7%)	1202.0/1284.0 (93.6%)	
Yes		103.0/1773.0 (5.8%)	21.0/489.0 (4.3%)	82.0/1284.0 (6.4%)	
Missing		22	5	17	
IBS^c^	1762/1795				0.013
No		1708.0/1762.0 (96.9%)	465.0/488.0 (95.3%)	1243.0/1274.0 (97.6%)	
Yes		54.0/1762.0 (3.1%)	23.0/488.0 (4.7%)	31.0/1274.0 (2.4%)	
Missing		33	6	27	
Cancer^c^	1768/1795				0.84
No		1604.0/1768.0 (90.7%)	442.0/486.0 (90.9%)	1162.0/1282.0 (90.6%)	
Yes		164.0/1768.0 (9.3%)	44.0/486.0 (9.1%)	120.0/1282.0 (9.4%)	
Missing		27	8	19	
Periodontits^c^	1765/1795				0.10
No		1348.0/1765.0 (76.4%)	352.0/478.0 (73.6%)	996.0/1287.0 (77.4%)	
Yes		417.0/1765.0 (23.6%)	126.0/478.0 (26.4%)	291.0/1287.0 (22.6%)	
Missing		30	16	14	
Rheumatoid arthritis^c^	1735/1795				0.68
No		1593.0/1735.0 (91.8%)	434.0/475.0 (91.4%)	1159.0/1260.0 (92.0%)	
Yes		142.0/1735.0 (8.2%)	41.0/475.0 (8.6%)	101.0/1260.0 (8.0%)	
Missing		60	19	41	
Regular use of medication^f^	1778/1795				< 0.001
No		555.0/1778.0 (31.2%)	68.0/488.0 (13.9%)	487.0/1290.0 (37.8%)	
Yes		1223.0/1778.0 (68.8%)	420.0/488.0 (86.1%)	803.0/1290.0 (62.2%)	
Missing		17	6	11	

^a^Median (IQR), Mean (SD) and Frequencies (N/%)

^b^Pearson’s Chi-squared test; Wilcoxon rank sum test; Fisher's exact test

^c^Self-reported

^d^A fasting blood sugar level from 100 to 125 mg/dL (5.6–7.0 mmol/L) is considered as prediabetes

^e^Self-reported diabetes mellitus type 2 and diagnosed by high basal glucose levels (a fasting blood sugar level of 126 mg/dL (7.0 mmol/L) or higher indicates diabetes mellitus type 2 diabetes)

^f^Self-reported and prescription list of the general practitioner

*MIG* metabolic inflammation group, *ROG* registration office group, *BMI* body mass index (BMI class: *UW* underweight, *NW* normal weight, *OW* overweight, *OBI* obesity grade I, *OBII* obesity grade II, *OBIII* obesity grade III), *BP* blood pressure, *HDL* high density lipoprotein, *LDL* low density lipoprotein, *CRP* C-reactive protein, *IL-6* interleukin 6, *T1DM* type 1 diabetes mellitus, *T2DM* type 2 diabetes mellitus, *IBD* inflammatory bowel disease and *IBS* irritable bowel syndrome

### Socioeconomic status and quality of life

More MIG subjects were current smokers than ROG subjects. Nearly one third of the ROG subjects finished school with university entrance qualification while only about 20% of MIG subjects completed university entrance qualification. MIG subjects in comparison to ROG subjects were more often unemployed, and only 8.1% of the MIG subjects reported being very content with their life (Table 4). Having no children was mostly reported by male MIG subjects (see Table S1).

### Medical conditions

One third of the MIG subjects had type 2 diabetes mellitus and related comorbidities e.g. high blood pressure or dyslipidaemia. Cardiac problems, liver and neurological diseases and allergic problems were more often reported by MIG subjects. More than 80% of the MIG subjects reported regular use of medication (Table 4). More female MIG subjects showed neurological diseases and IBS whereas male MIG subjects showed a higher prevalence of periodontitis (see Table S1).

### Activity and nutrition behaviour

Complete data on activity and nutrition behaviour from 1670 subjects were available. The FFQ was completed only partially or not at all by 125 subjects. ROG subjects watched less TV and were more active in general than MIG subjects. ROG subjects, however, consumed a high-fat, low-fibre diet, as did MIG subjects. MIG subjects consumed significantly less alcohol than ROG subjects, but more minerals and organic acids (Table 5). This was also true after separating MIG and ROG subjects by sex (see Table S2).

**Table 5:** Activity and nutrition parameters of the FoCus cohort subjects at baseline stratified by type of recruitment

Characteristics	N	Overall, N = 1670^a^	MIG, N = 450^a^	ROG, N = 1220^a^	*p*-Value^b^
Sex	1670/1670				< 0.001
Females		1050.0/1670.0 (62.9%)	334.0/450.0 (74.2%)	716.0/1220.0 (58.7%)	
Males		620.0/1670.0 (37.1%)	116.0/450.0 (25.8%)	504.0/1220.0 (41.3%)	
TV-watching (h/day)	1670/1670	3.0 (1.5, 4.0)	3.0 (2.0, 5.0)	2.0 (1.5, 3.0)	< 0.001
Missing		0	0	0	
Daily activity (min./week)	1670/1670	915.0 (540.0, 1500.0)	885.0 (495.0, 1410.0)	945.0 (547.5, 1530.0)	0.10
Missing		0	0	0	
Sports activity (min./week)	1670/1670	210.0 (60.0, 375.0)	93.8 (0.0, 253.1)	240.0 (105.0, 420.0)	< 0.001
Missing		0	0	0	
Night sleep (h/night)	1670/1670	7.0 (6.0, 8.0)	7.0 (6.0, 8.0)	7.0 (7.0, 8.0)	0.005
Missing		0	0	0	
energy (kJ/day)	1670/1670	8732.0 (7148.9 10,782.4)	8522.0 (6953.0 11,141.2)	8764.1 (7210.8 10,722.3)	0.38
Missing		0	0	0	
Carbohydrates (% energy)	1670/1670	41.6 (38.3, 45.6)	42.4 (38.7, 46.6)	41.4 (37.9, 45.3)	< 0.001
Missing		0	0	0	
Proteins (% energy)	1670/1670	14.6 (13.3, 16.0)	15.3 (14.0, 16.8)	14.4 (13.1, 15.8)	< 0.001
Missing		0	0	0	
Fats (% energy)	1670/1670	39.6 (36.1, 42.8)	39.7 (35.5, 43.0)	39.6 (36.3, 42.6)	0.59
Missing		0	0	0	
Fibres (g/day)	1670/1670	21.8 (18.8, 25.3)	22.0 (18.6, 25.5)	21.8 (18.9, 25.2)	0.89
Missing		0	0	0	
Organic acids (g/day)	1670/1670	7.4 (5.8, 9.1)	7.7 (6.3, 9.6)	7.3 (5.6, 8.9)	< 0.001
Missing		0	0	0	
Minerals (g/day)	1670/1670	16.7 (15.6, 18.0)	17.1 (15.6, 18.3)	16.6 (15.6, 17.8)	0.002
Missing		0	0	0	
Alcohol intake (g/day)	1670/1670	1.9 (0.7, 4.4)	0.7 (0.4, 2.0)	2.6 (1.0, 5.1)	< 0.001
Missing		0	0	0	
Salt intake (g/day)	1670/1670	5.6 (5.1, 6.2)	5.8 (5.3, 6.6)	5.5 (5.0, 6.1)	< 0.001
Missing		0	0	0	

^a^n/N (%); Median (IQR)

^b^Pearson’s Chi-squared test; Wilcoxon rank sum test

*MIG* metabolic inflammation group, *ROG* registration office group, *h* hours, *min.* minutes

### Follow‑up 1 examination

After five years, 820 subjects (n = 620 ROG and n = 200 MIG subjects) with an overall median age of 61.0 years (IQR: 52.0; 71.0) and median BMI of 26.7 kg/m^2^ (IQR: 23.6; 32.3) took part in the first follow-up examination.

Responders included more subjects from ROG, with no sex differences between responders and non-responders. Responders were older than non-responders and were more often retired at baseline. By contrast more responders reported high blood pressure or dyslipidaemia at baseline (see Table S3).

For first follow-up analyses, incident diabetes and changes in BMI status were available for 817 and 810 subjects respectively. During the follow-up period, 8% (n = 16) of 198 MIG subjects and 2.1% (n = 13) of 619 ROG subjects developed new type 2 diabetes mellitus. Most of the ROG subjects of normal weight at baseline remained in the normal weight group (84.8%), 0.7% changed to the underweight group, while a larger number developed overweight (12.3%). Nearly half (43.4%) of the MIG subjects with morbid obesity remained in obesity class III and 45.9% changed into lower obesity classes. The reason might be that subjects with obesity class II/III were incorporated into various dietary and surgery programs at the UKSH during the 5 years’ before follow up. Three percent and 11.5% of the MIG subjects were incorporated into the diet and surgery programs respectively at the UKSH.

### Findings to date

Data from the FoCus cohort were used to address several different research questions to date, relating to, e.g., diabetes and microbiome [32–34], microbiome association studies [35], multiple sclerosis [36], hypertension and genome wide association studies [37–40] in national and international consortia as well as in small more specific research [41–47] and intervention studies [48] (see Table S4).

Key publications resulting from these studies:

- In a translational human study, *Parasutterella sp.* was assessed in the FoCus cohort followed by a validation of major results in an independent Canadian cohort. Additionally, *Parasutterella sp.* abundance was examined in response to a weight loss intervention (n = 55). *Parasutterella sp*. was positively associated with BMI and type 2 diabetes mellitus independently of the reduced microbiome α/β diversity and low-grade inflammation commonly found in obesity. High *Parasutterella sp.* abundance was associated with a reduction in L-cysteine linking *Parasutterella sp.* to type 2 diabetes and obesity development [35].
- In two previous analyses from Barberesko et al. [41, 49], dietary patterns of FoCus cohort participants were related to metabolic syndrome and inflammation. In a first publication [49] two similar dietary patterns were characterized representing a ‘traditional German diet’ (potatoes, legumes, cabbage, other vegetables, pork, beef, processed meat, sauce, other fats and bouillon) which were positive associated with BMI, waist circumference, the metabolic syndrome as well as with anthropometric measures and biomarkers [49]. In a second publication, Barbaresko et al. [41] showed that a pro-inflammatory dietary pattern was characterized by high intakes of soft drinks, meat, potatoes, sauces, and low intakes of cereals, wine, nuts and seeds, vegetarian dishes, vegetable oil, and fish products.
- Wingless-Type MMTV Integration Site Family, Member 5A (WNT5A) plays a critical role in normal cellular processes (e.g. cell proliferation, migration, and differentiation) and is implicated in metabolic inflammation in rodent models. WNT5A was analysed in a FoCus cohort subgroup to gain deeper insights into WNT5A physiology in humans. WNT5A levels were significantly positively correlated to IL-6 and triglyceride levels and, in diabetes, to fasting plasma glucose levels. These levels were not influenced by common single-nucleotide polymorphisms. In addition, WNT5A levels were decreased in subjects with higher intake of the long-chain eicosatetraenoic acid and high gut microbiome α diversity [45].
- Secreted frizzled‐related protein 5 (sFRP 5) serum levels in human periodontitis were investigated in a nested case–control study. In this project periodontitis was used as model of metabolic inflammation induced by unfavourable nutrition. Schulz et al. [47] used a nested case–control study including patients with periodontitis and tooth loss as well as patients with periodontitis without tooth loss and matched individuals from the FoCus cohort. When compared to patients with periodontitis without tooth loss and matched controls patients with periodontitis and tooth loss had significantly lower sFRP5 serum levels.
- Kreutzer et al. [43] analysed data from obese and matched non-obese subjects from the FoCus cohort with regard to the relationship between inflammation in the appetite regulating hypothalamus and obesity. The medio basal hypothalamus (MBH) and the T2 hyperintensity as a measure of hypothalamic inflammation (HI) were assessed by magnetic resonance imaging (MRI). In obese subjects, T2 hyperintensity was found in the left but not the right MBH and strongly linked to systemic low-grade inflammation. Nutritional analysis and 16S rDNA microbiome sequencing were performed. Of interest, no direct effect of dietary components on HI was found but it became evident that a high-fat diet seems to induce a decrease in specific gut bacteria [43].
- In specific subgroups, two nutrition intervention studies were performed. The first study was a targeted microbiome intervention study with microencapsulated delayed-release niacin which beneficially affects insulin sensitivity in humans [42]. There were no systemic side effects. Favourable microbiome changes induced by microencapsulated delayed-release niacin were associated with an improvement of biomarkers for systemic insulin sensitivity and metabolic inflammation. The second study was a double-blind cross-over intervention study with a whey drink supplemented (± β-casein lysate). Primary outcomes of the study were inflammation biomarkers e.g. IL-6 and CRP. There was no effect on inflammation, but the serum level of fibroblast growth factor 21 (FGF-21) which is associated with beneficial effects (e.g. glucose-lowering and improvement of insulin sensitivity) was increased in the verum group [48].

## Future perspectives of the FoCus cohort

In the future, more biomarkers for the identification of metabolic inflammation will be investigated. Furthermore, it is planned to conduct targeted research on the topic of "microbiome-centred research" on the basis of a previous work on niacin [42]. The availability of different omics data will enable us to perform complex and state-of-the-art multi-omics statistics in the FoCus cohort regarding different research questions cross-sectionally and longitudinally. These will extend the research in metabolic inflammation to the nutrition-gut-microbiome-host-metabolism axis.

## Strengths and limitations

The FoCus cohort study is a partly population-based longitudinal study in northern Germany with a broad range of health, nutrition, genetic, microbiome and metabolome data. A wide range of assessed data enable comprehensive longitudinal analyses of health trajectories and their determinants. The data included results of health interviews which are supplemented with blood and stool sample collection. For the follow-up, the data collection changed from invited visits at the study centre to visits at the general practitioner. Possible effects regarding this change must be carefully considered for further analyses. For example, the collection technique for blood sampling may differ, but the postal dispatch of serum and stool samples is common practice for laboratories in Germany. The dispatch times are considered accordingly during the evaluation of data on the basis of an empirical scatter and a permitted scatter range for individual analyses. Self-reporting of weight and height is also common practice in epidemiological studies, and studies showed that participants reporting their height and weight with reasonable accuracy suggesting that BMI derived from self-reported height and weight is a valid measure. A further limitation during the next years could be a relatively high dropout rate. This could lead to a selection bias towards participants with a special health interest. Using a longitudinal weighting factor could be included in further analyses to diminish possible effects of selective study participation.

## Supplementary Information

Below is the link to the electronic supplementary material.

Supplementary file1 (DOCX 38 kb)

Supplementary file2 (DOCX 19 kb)

Supplementary file3 (DOCX 41 kb)

Supplementary file4 (DOCX 60 kb)
