# Supplementary material for: Cohort profile: the Food Chain Plus (FoCus) cohort
Source: Eur J Epidemiol. 2022 Oct 16;37(10):1087–105. doi: 10.1007/s10654-022-00924-y (PMC9630232; doi:10.1007/s10654-022-00924-y)
Supplement: Supplementary file 1 — Supplementary file1 (DOCX 38 kb) [file 10654_2022_924_MOESM1_ESM.docx]

**Table S1** Demographic, clinical and laboratory characteristics of the FoCus cohort subjects at baseline stratified by sex and type of recruitment

|  | **Females** | | | | | | **Males** | | | | |
| --- | --- | --- | --- | --- | --- | --- | --- | --- | --- | --- | --- |
|  | | N | Overall, N = 1,131^1^ | MIG, N = 370^1^ | ROG, N = 761^1^ | p-value^2^ | N | Overall, N = 664^1^ | MIG, N = 124^1^ | ROG, N = 540^1^ | p-value^2^ |
| **Age (years)** | | 1,131 / 1,131 | 50.0 (40.5, 61.0) | 47.5 (38.0, 56.0) | 52.0 (42.0, 64.0) | <0.001 | 664 / 664 | 55.0 (46.0, 65.0) | 51.0 (43.0, 58.2) | 56.0 (46.0, 67.0) | <0.001 |
| Missing | |  | 0 | 0 | 0 |  |  | 0 | 0 | 0 |  |
| **Height (cm)** | | 1,131 / 1,131 | 168.0 (163.0, 172.0) | 168.0 (163.0, 172.0) | 168.0 (163.0, 172.0) | 0.50 | 664 / 664 | 180.0 (176.0, 185.0) | 182.0 (177.5, 186.0) | 180.0 (175.9, 184.0) | 0.014 |
| Missing | |  | 0 | 0 | 0 |  |  | 0 | 0 | 0 |  |
| **Weight (kg)** | | 1,131 / 1,131 | 78.1 (64.8, 106.1) | 119.9 (101.0, 137.3) | 68.6 (62.0, 79.0) | <0.001 | 664 / 664 | 89.4 (79.3, 103.9) | 139.8 (112.6, 166.0) | 86.6 (77.4, 96.0) | <0.001 |
| Missing | |  | 0 | 0 | 0 |  |  | 0 | 0 | 0 |  |
| **BMI (kg/m²)** | | 1,131 / 1,131 | 27.9 (23.1, 38.7) | 43.1 (36.8, 48.5) | 24.3 (22.0, 28.0) | <0.001 | 664 / 664 | 27.5 (24.7, 32.1) | 42.1 (35.9, 49.7) | 26.5 (24.2, 29.3) | <0.001 |
| Range | | 1,131 / 1,131 | 14.5, 83.2 | 19.0, 70.4 | 14.5, 83.2 |  |  | 15.8, 72.7 | 22.2, 72.7 | 15.8, 54.8 |  |
| Missing | |  | 0 | 0 | 0 |  |  | 0 | 0 | 0 |  |
| **BMI class** | | 1,131 / 1,131 |  |  |  | <0.001 | 664 / 664 |  |  |  |  |
| UW  (< 18.5 kg/m²) | |  | 21.0 / 1,131.0 (1.9%) | 0.0 / 370.0 (0.0%) | 21.0 / 761.0 (2.8%) |  |  | 3.0 / 664.0 (0.5%) | 0.0 / 124.0 (0.0%) | 3.0 / 540.0 (0.6%) |  |
| NW  (18.5 to 24.9 kg/m²) | |  | 396.0 / 1,131.0 (35.0%) | 3.0 / 370.0 (0.8%) | 393.0 / 761.0 (51.6%) |  |  | 174.0 / 664.0 (26.2%) | 2.0 / 124.0 (1.6%) | 172.0 / 540.0 (31.9%) |  |
| OW  (25.0 to 29.9 kg/m²) | |  | 224.0 / 1,131.0 (19.8%) | 15.0 / 370.0 (4.1%) | 209.0 / 761.0 (27.5%) |  |  | 259.0 / 664.0 (39.0%) | 10.0 / 124.0 (8.1%) | 249.0 / 540.0 (46.1%) |  |
| OBI  (30.0 to 34.9 kg/m²) | |  | 142.0 / 1,131.0 (12.6%) | 55.0 / 370.0 (14.9%) | 87.0 / 761.0 (11.4%) |  |  | 98.0 / 664.0 (14.8%) | 17.0 / 124.0 (13.7%) | 81.0 / 540.0 (15.0%) |  |
| OBII (35.0 to 39.9 kg/m²) | |  | 93.0 / 1,131.0 (8.2%) | 64.0 / 370.0 (17.3%) | 29.0 / 761.0 (3.8%) |  |  | 47.0 / 664.0 (7.1%) | 19.0 / 124.0 (15.3%) | 28.0 / 540.0 (5.2%) |  |
| OBIII  (≥ 40.0 kg/m²) | |  | 255.0 / 1,131.0 (22.5%) | 233.0 / 370.0 (63.0%) | 22.0 / 761.0 (2.9%) |  |  | 83.0 / 664.0 (12.5%) | 76.0 / 124.0 (61.3%) | 7.0 / 540.0 (1.3%) |  |
| Missing | |  | 0 | 0 | 0 |  |  | 0 | 0 | 0 |  |
| **Hip-circum-ference (cm)** | | 1,026 / 1,131 | 109.0 (101.0, 123.0) | 132.0 (123.0, 140.0) | 104.0 (99.0, 111.0) | <0.001 | 613 / 664 | 107.0 (102.0, 113.0) | 119.5 (112.8, 130.0) | 106.3 (102.0, 111.8) | <0.001 |
| Missing | |  | 105 | 94 | 11 |  |  | 51 | 48 | 3 |  |
| **Waist-circum-ference (cm)** | | 1,052 / 1,131 | 92.0 (79.9, 113.0) | 122.0 (112.0, 133.0) | 85.0 (76.0, 96.0) | <0.001 | 608 / 664 | 101.0 (93.0, 112.0) | 128.0 (113.5, 138.0) | 99.0 (92.0, 108.0) | <0.001 |
| Missing | |  | 79 | 73 | 6 |  |  | 56 | 51 | 5 |  |
| **BP systolic (mmHg)** | | 1,131 / 1,131 | 126.8 (12.2) | 132.8 (10.8) | 123.9 (11.7) | <0.001 | 664 / 664 | 133.0 (10.7) | 136.6 (10.7) | 132.2 (10.5) | <0.001 |
| Missing | |  | 0 | 0 | 0 |  |  | 0 | 0 | 0 |  |
| **BP diastolic (mmHg)** | | 1,131 / 1,131 | 79.7 (7.0) | 82.9 (6.7) | 78.1 (6.6) | <0.001 | 664 / 664 | 81.6 (6.0) | 83.3 (6.7) | 81.3 (5.7) | <0.001 |
| Missing | |  | 0 | 0 | 0 |  |  | 0 | 0 | 0 |  |
| **Tri-glycerides (mg/dL)** | | 1,128 / 1,131 | 103.0 (73.0, 145.2) | 134.0 (98.8, 182.0) | 90.0 (66.0, 126.0) | <0.001 | 662 / 664 | 117.0 (82.0, 171.0) | 157.0 (113.5, 229.0) | 110.0 (77.0, 157.5) | <0.001 |
| Missing | |  | 3 | 2 | 1 |  |  | 2 | 1 | 1 |  |
| **Cholesterol total (mmol/L)** | | 631 / 1,131 | 4.7 (4.1, 5.3) | 4.7 (4.1, 5.2) | 4.6 (4.1, 5.3) | 0.84 | 336 / 664 | 4.4 (3.8, 4.9) | 4.1 (3.6, 4.6) | 4.5 (3.8, 5.0) | 0.003 |
| Missing | |  | 500 | 106 | 394 |  |  | 328 | 29 | 299 |  |
| **LDL-cholesterol (mmol/L)** | | 62 / 1,131 | 3.1 (2.6, 3.7) | NA (NA, NA) | 3.1 (2.6, 3.7) |  | 61 / 664 | 3.1 (2.5, 3.8) | NA (NA, NA) | 3.1 (2.5, 3.8) |  |
| Missing | |  | 1,069 | 370 | 699 |  |  | 603 | 124 | 479 |  |
| **HDL-cholesterol (mmol/L)** | | 62 / 1,131 | 1.6 (1.4, 1.9) | NA (NA, NA) | 1.6 (1.4, 1.9) |  | 61 / 664 | 1.4 (1.2, 1.8) | NA (NA, NA) | 1.4 (1.2, 1.8) |  |
| Missing | |  | 1,069 | 370 | 699 |  |  | 603 | 124 | 479 |  |
| **Lipoprotein a (mg/L)** | | 438 / 1,131 | 250.5 (142.0, 493.2) | 238.5 (149.5, 473.8) | 262.0 (135.2, 495.5) | 0.80 | 237 / 664 | 264.0 (155.0, 485.0) | 325.0 (202.0, 582.0) | 243.0 (145.0, 446.8) | 0.021 |
| Missing | |  | 693 | 190 | 503 |  |  | 427 | 73 | 354 |  |
| **Glucose (mg/dL)** | | 1,127 / 1,131 | 93.0 (87.0, 102.0) | 99.0 (90.0, 112.0) | 91.0 (86.0, 98.0) | <0.001 | 662 / 664 | 99.0 (91.0, 109.0) | 106.0 (98.0, 130.5) | 97.0 (91.0, 105.0) | <0.001 |
| Missing | |  | 4 | 2 | 2 |  |  | 2 | 1 | 1 |  |
| **Insulin (mU/L)** | | 1,120 / 1,131 | 10.0 (6.6, 16.9) | 18.0 (11.0, 28.4) | 8.1 (5.7, 11.6) | <0.001 | 658 / 664 | 10.5 (7.0, 19.9) | 26.5 (15.0, 42.7) | 9.3 (6.5, 15.3) | <0.001 |
| Missing | |  | 11 | 5 | 6 |  |  | 6 | 2 | 4 |  |
| **HOMA-IR** | | 1,123 / 1,131 | 2.3 (1.5, 4.1) | 4.4 (2.6, 8.2) | 1.8 (1.3, 2.7) | <0.001 | 662 / 664 | 2.6 (1.6, 5.1) | 7.0 (3.9, 12.3) | 2.3 (1.5, 3.8) | <0.001 |
| Missing | |  | 8 | 4 | 4 |  |  | 2 | 1 | 1 |  |
| **CRP (mg/L)** | | 797 / 1,131 | 3.5 (1.8, 7.5) | 6.8 (3.5, 11.8) | 2.3 (1.4, 4.0) | <0.001 | 412 / 664 | 2.9 (1.5, 5.1) | 4.9 (3.2, 9.4) | 2.1 (1.3, 3.9) | <0.001 |
| Missing | |  | 334 | 19 | 315 |  |  | 252 | 12 | 240 |  |
| **IL-6 (pg/mL)** | | 900 / 1,131 | 3.8 (2.6, 5.5) | 4.7 (3.5, 6.6) | 3.3 (2.3, 4.7) | <0.001 | 553 / 664 | 3.7 (2.7, 5.4) | 5.4 (3.7, 7.8) | 3.5 (2.5, 4.9) | <0.001 |
| Missing | |  | 231 | 28 | 203 |  |  | 111 | 9 | 102 |  |
| **Smoking habits** | | 1,088 / 1,131 |  |  |  | 0.001 | 656 / 664 |  |  |  | 0.83 |
| Never smoking | |  | 438.0 / 1,088.0 (40.3%) | 110.0 / 347.0 (31.7%) | 328.0 / 741.0 (44.3%) |  |  | 189.0 / 656.0 (28.8%) | 33.0 / 123.0 (26.8%) | 156.0 / 533.0 (29.3%) |  |
| Previous smoking | |  | 372.0 / 1,088.0 (34.2%) | 133.0 / 347.0 (38.3%) | 239.0 / 741.0 (32.3%) |  |  | 301.0 / 656.0 (45.9%) | 61.0 / 123.0 (49.6%) | 240.0 / 533.0 (45.0%) |  |
| Less than 3 months | |  | 75.0 / 1,088.0 (6.9%) | 27.0 / 347.0 (7.8%) | 48.0 / 741.0 (6.5%) |  |  | 49.0 / 656.0 (7.5%) | 8.0 / 123.0 (6.5%) | 41.0 / 533.0 (7.7%) |  |
| Smoking | |  | 203.0 / 1,088.0 (18.7%) | 77.0 / 347.0 (22.2%) | 126.0 / 741.0 (17.0%) |  |  | 117.0 / 656.0 (17.8%) | 21.0 / 123.0 (17.1%) | 96.0 / 533.0 (18.0%) |  |
| Missing | |  | 43 | 23 | 20 |  |  | 8 | 1 | 7 |  |
| **School education** | | 1,122 / 1,131 |  |  |  |  | 662 / 664 |  |  |  | <0.001 |
| University qualification | |  | 319.0 / 1,122.0 (28.4%) | 58.0 / 365.0 (15.9%) | 261.0 / 757.0 (34.5%) |  |  | 224.0 / 662.0 (33.8%) | 26.0 / 122.0 (21.3%) | 198.0 / 540.0 (36.7%) |  |
| Technical college quali-fication | |  | 100.0 / 1,122.0 (8.9%) | 32.0 / 365.0 (8.8%) | 68.0 / 757.0 (9.0%) |  |  | 96.0 / 662.0 (14.5%) | 10.0 / 122.0 (8.2%) | 86.0 / 540.0 (15.9%) |  |
| Middle school | |  | 435.0 / 1,122.0 (38.8%) | 148.0 / 365.0 (40.5%) | 287.0 / 757.0 (37.9%) |  |  | 178.0 / 662.0 (26.9%) | 36.0 / 122.0 (29.5%) | 142.0 / 540.0 (26.3%) |  |
| Secondary school | |  | 257.0 / 1,122.0 (22.9%) | 120.0 / 365.0 (32.9%) | 137.0 / 757.0 (18.1%) |  |  | 160.0 / 662.0 (24.2%) | 47.0 / 122.0 (38.5%) | 113.0 / 540.0 (20.9%) |  |
| No degree | |  | 11.0 / 1,122.0 (1.0%) | 7.0 / 365.0 (1.9%) | 4.0 / 757.0 (0.5%) |  |  | 4.0 / 662.0 (0.6%) | 3.0 / 122.0 (2.5%) | 1.0 / 540.0 (0.2%) |  |
| Missing | |  | 9 | 5 | 4 |  |  | 2 | 2 | 0 |  |
| **Em-ployment** | | 1,119 / 1,131 |  |  |  | <0.001 | 664 / 664 |  |  |  | <0.001 |
| Full time | |  | 279.0 / 1,119.0 (24.9%) | 96.0 / 367.0 (26.2%) | 183.0 / 752.0 (24.3%) |  |  | 324.0 / 664.0 (48.8%) | 51.0 / 124.0 (41.1%) | 273.0 / 540.0 (50.6%) |  |
| Part time | |  | 317.0 / 1,119.0 (28.3%) | 80.0 / 367.0 (21.8%) | 237.0 / 752.0 (31.5%) |  |  | 37.0 / 664.0 (5.6%) | 6.0 / 124.0 (4.8%) | 31.0 / 540.0 (5.7%) |  |
| Un-employed | |  | 69.0 / 1,119.0 (6.2%) | 48.0 / 367.0 (13.1%) | 21.0 / 752.0 (2.8%) |  |  | 39.0 / 664.0 (5.9%) | 20.0 / 124.0 (16.1%) | 19.0 / 540.0 (3.5%) |  |
| Retired | |  | 305.0 / 1,119.0 (27.3%) | 88.0 / 367.0 (24.0%) | 217.0 / 752.0 (28.9%) |  |  | 219.0 / 664.0 (33.0%) | 34.0 / 124.0 (27.4%) | 185.0 / 540.0 (34.3%) |  |
| other | |  | 149.0 / 1,119.0 (13.3%) | 55.0 / 367.0 (15.0%) | 94.0 / 752.0 (12.5%) |  |  | 45.0 / 664.0 (6.8%) | 13.0 / 124.0 (10.5%) | 32.0 / 540.0 (5.9%) |  |
| Missing | |  | 12 | 3 | 9 |  |  | 0 | 0 | 0 |  |
| **Content of life** | | 1,109 / 1,131 |  |  |  | <0.001 | 655 / 664 |  |  |  | <0.001 |
| Very content | |  | 240.0 / 1,109.0 (21.6%) | 27.0 / 357.0 (7.6%) | 213.0 / 752.0 (28.3%) |  |  | 163.0 / 655.0 (24.9%) | 12.0 / 122.0 (9.8%) | 151.0 / 533.0 (28.3%) |  |
| Content | |  | 601.0 / 1,109.0 (54.2%) | 153.0 / 357.0 (42.9%) | 448.0 / 752.0 (59.6%) |  |  | 358.0 / 655.0 (54.7%) | 40.0 / 122.0 (32.8%) | 318.0 / 533.0 (59.7%) |  |
| Not so content | |  | 222.0 / 1,109.0 (20.0%) | 139.0 / 357.0 (38.9%) | 83.0 / 752.0 (11.0%) |  |  | 99.0 / 655.0 (15.1%) | 46.0 / 122.0 (37.7%) | 53.0 / 533.0 (9.9%) |  |
| Not at all content | |  | 46.0 / 1,109.0 (4.1%) | 38.0 / 357.0 (10.6%) | 8.0 / 752.0 (1.1%) |  |  | 35.0 / 655.0 (5.3%) | 24.0 / 122.0 (19.7%) | 11.0 / 533.0 (2.1%) |  |
| Missing | |  | 22 | 13 | 9 |  |  | 9 | 2 | 7 |  |
| **Household type** | | 1,119 / 1,131 |  |  |  | 0.001 | 661 / 664 |  |  |  | 0.005 |
| Living with partner | |  | 775.0 / 1,119.0 (69.3%) | 230.0 / 369.0 (62.3%) | 545.0 / 750.0 (72.7%) |  |  | 539.0 / 661.0 (81.5%) | 88.0 / 123.0 (71.5%) | 451.0 / 538.0 (83.8%) |  |
| Living alone | |  | 260.0 / 1,119.0 (23.2%) | 101.0 / 369.0 (27.4%) | 159.0 / 750.0 (21.2%) |  |  | 96.0 / 661.0 (14.5%) | 29.0 / 123.0 (23.6%) | 67.0 / 538.0 (12.5%) |  |
| Other types | |  | 84.0 / 1,119.0 (7.5%) | 38.0 / 369.0 (10.3%) | 46.0 / 750.0 (6.1%) |  |  | 26.0 / 661.0 (3.9%) | 6.0 / 123.0 (4.9%) | 20.0 / 538.0 (3.7%) |  |
| Missing | |  | 12 | 1 | 11 |  |  | 3 | 1 | 2 |  |
| **Children** | | 1,125 / 1,131 |  |  |  | 0.075 | 660 / 664 |  |  |  | 0.002 |
| no | |  | 355.0 / 1,125.0 (31.6%) | 129.0 / 367.0 (35.1%) | 226.0 / 758.0 (29.8%) |  |  | 195.0 / 660.0 (29.5%) | 52.0 / 122.0 (42.6%) | 143.0 / 538.0 (26.6%) |  |
| yes | |  | 770.0 / 1,125.0 (68.4%) | 238.0 / 367.0 (64.9%) | 532.0 / 758.0 (70.2%) |  |  | 463.0 / 660.0 (70.2%) | 70.0 / 122.0 (57.4%) | 393.0 / 538.0 (73.0%) |  |
| unknown | |  | 0.0 / 1,125.0 (0.0%) | 0.0 / 367.0 (0.0%) | 0.0 / 758.0 (0.0%) |  |  | 2.0 / 660.0 (0.3%) | 0.0 / 122.0 (0.0%) | 2.0 / 538.0 (0.4%) |  |
| Missing | |  | 6 | 3 | 3 |  |  | 4 | 2 | 2 |  |
| **Diabetes^3^** | | 1,128 / 1,131 |  |  |  | <0.001 | 663 / 664 |  |  |  | <0.001 |
| Normal | |  | 751.0 / 1,128.0 (66.6%) | 171.0 / 369.0 (46.3%) | 580.0 / 759.0 (76.4%) |  |  | 335.0 / 663.0 (50.5%) | 26.0 / 124.0 (21.0%) | 309.0 / 539.0 (57.3%) |  |
| Pre-diabetes^4^ | |  | 213.0 / 1,128.0 (18.9%) | 87.0 / 369.0 (23.6%) | 126.0 / 759.0 (16.6%) |  |  | 206.0 / 663.0 (31.1%) | 37.0 / 124.0 (29.8%) | 169.0 / 539.0 (31.4%) |  |
| T1DM | |  | 4.0 / 1,128.0 (0.4%) | 2.0 / 369.0 (0.5%) | 2.0 / 759.0 (0.3%) |  |  | 4.0 / 663.0 (0.6%) | 0.0 / 124.0 (0.0%) | 4.0 / 539.0 (0.7%) |  |
| T2DM^5^ | |  | 138.0 / 1,128.0 (12.2%) | 97.0 / 369.0 (26.3%) | 41.0 / 759.0 (5.4%) |  |  | 115.0 / 663.0 (17.3%) | 60.0 / 124.0 (48.4%) | 55.0 / 539.0 (10.2%) |  |
| other | |  | 22.0 / 1,128.0 (2.0%) | 12.0 / 369.0 (3.3%) | 10.0 / 759.0 (1.3%) |  |  | 3.0 / 663.0 (0.5%) | 1.0 / 124.0 (0.8%) | 2.0 / 539.0 (0.4%) |  |
| Missing | |  | 3 | 1 | 2 |  |  | 1 | 0 | 1 |  |
| **Hyper-tension^3^** | | 1,119 / 1,131 |  |  |  | <0.001 | 655 / 664 |  |  |  | <0.001 |
| no | |  | 662.0 / 1,119.0 (59.2%) | 131.0 / 366.0 (35.8%) | 531.0 / 753.0 (70.5%) |  |  | 361.0 / 655.0 (55.1%) | 28.0 / 119.0 (23.5%) | 333.0 / 536.0 (62.1%) |  |
| yes | |  | 457.0 / 1,119.0 (40.8%) | 235.0 / 366.0 (64.2%) | 222.0 / 753.0 (29.5%) |  |  | 294.0 / 655.0 (44.9%) | 91.0 / 119.0 (76.5%) | 203.0 / 536.0 (37.9%) |  |
| Missing | |  | 12 | 4 | 8 |  |  | 9 | 5 | 4 |  |
| **Dys-lipidemia^3^** | | 1,104 / 1,131 |  |  |  | 0.005 | 651 / 664 |  |  |  | 0.020 |
| no | |  | 808.0 / 1,104.0 (73.2%) | 245.0 / 361.0 (67.9%) | 563.0 / 743.0 (75.8%) |  |  | 426.0 / 651.0 (65.4%) | 67.0 / 119.0 (56.3%) | 359.0 / 532.0 (67.5%) |  |
| yes | |  | 296.0 / 1,104.0 (26.8%) | 116.0 / 361.0 (32.1%) | 180.0 / 743.0 (24.2%) |  |  | 225.0 / 651.0 (34.6%) | 52.0 / 119.0 (43.7%) | 173.0 / 532.0 (32.5%) |  |
| Missing | |  | 27 | 9 | 18 |  |  | 13 | 5 | 8 |  |
| **Myocardial infarction^3^** | | 1,126 / 1,131 |  |  |  | 0.19 | 655 / 664 |  |  |  | 0.40 |
| no | |  | 1,115.0 / 1,126.0 (99.0%) | 360.0 / 366.0 (98.4%) | 755.0 / 760.0 (99.3%) |  |  | 612.0 / 655.0 (93.4%) | 111.0 / 121.0 (91.7%) | 501.0 / 534.0 (93.8%) |  |
| yes | |  | 11.0 / 1,126.0 (1.0%) | 6.0 / 366.0 (1.6%) | 5.0 / 760.0 (0.7%) |  |  | 43.0 / 655.0 (6.6%) | 10.0 / 121.0 (8.3%) | 33.0 / 534.0 (6.2%) |  |
| Missing | |  | 5 | 4 | 1 |  |  | 9 | 3 | 6 |  |
| **Cardiac failure^3^** | | 1,111 / 1,131 |  |  |  | 0.039 | 650 / 664 |  |  |  | <0.001 |
| no | |  | 1,076.0 / 1,111.0 (96.8%) | 344.0 / 361.0 (95.3%) | 732.0 / 750.0 (97.6%) |  |  | 625.0 / 650.0 (96.2%) | 106.0 / 120.0 (88.3%) | 519.0 / 530.0 (97.9%) |  |
| yes | |  | 35.0 / 1,111.0 (3.2%) | 17.0 / 361.0 (4.7%) | 18.0 / 750.0 (2.4%) |  |  | 25.0 / 650.0 (3.8%) | 14.0 / 120.0 (11.7%) | 11.0 / 530.0 (2.1%) |  |
| Missing | |  | 20 | 9 | 11 |  |  | 14 | 4 | 10 |  |
| **Liver disease^3^** | | 1,117 / 1,131 |  |  |  | 0.048 | 656 / 664 |  |  |  | <0.001 |
| no | |  | 1,053.0 / 1,117.0 (94.3%) | 335.0 / 363.0 (92.3%) | 718.0 / 754.0 (95.2%) |  |  | 611.0 / 656.0 (93.1%) | 99.0 / 119.0 (83.2%) | 512.0 / 537.0 (95.3%) |  |
| yes | |  | 64.0 / 1,117.0 (5.7%) | 28.0 / 363.0 (7.7%) | 36.0 / 754.0 (4.8%) |  |  | 45.0 / 656.0 (6.9%) | 20.0 / 119.0 (16.8%) | 25.0 / 537.0 (4.7%) |  |
| Missing | |  | 14 | 7 | 7 |  |  | 8 | 5 | 3 |  |
| **Neuro-logical disease^3^** | | 1,094 / 1,131 |  |  |  | <0.001 | 650 / 664 |  |  |  | 0.069 |
| no | |  | 911.0 / 1,094.0 (83.3%) | 269.0 / 356.0 (75.6%) | 642.0 / 738.0 (87.0%) |  |  | 597.0 / 650.0 (91.8%) | 108.0 / 123.0 (87.8%) | 489.0 / 527.0 (92.8%) |  |
| yes | |  | 183.0 / 1,094.0 (16.7%) | 87.0 / 356.0 (24.4%) | 96.0 / 738.0 (13.0%) |  |  | 53.0 / 650.0 (8.2%) | 15.0 / 123.0 (12.2%) | 38.0 / 527.0 (7.2%) |  |
| Missing | |  | 37 | 14 | 23 |  |  | 14 | 1 | 13 |  |
| **Stroke^3^** | | 1,123 / 1,131 |  |  |  | 0.35 | 659 / 664 |  |  |  | 0.33 |
| no | |  | 1,107.0 / 1,123.0 (98.6%) | 362.0 / 369.0 (98.1%) | 745.0 / 754.0 (98.8%) |  |  | 643.0 / 659.0 (97.6%) | 122.0 / 123.0 (99.2%) | 521.0 / 536.0 (97.2%) |  |
| yes | |  | 16.0 / 1,123.0 (1.4%) | 7.0 / 369.0 (1.9%) | 9.0 / 754.0 (1.2%) |  |  | 16.0 / 659.0 (2.4%) | 1.0 / 123.0 (0.8%) | 15.0 / 536.0 (2.8%) |  |
| Missing | |  | 8 | 1 | 7 |  |  | 5 | 1 | 4 |  |
| **Respiratory disease^3^** | | 265 / 1,131 |  |  |  | 0.041 | 125 / 664 |  |  |  | 0.007 |
| Asthma | |  | 106.0 / 265.0 (40.0%) | 58.0 / 136.0 (42.6%) | 48.0 / 129.0 (37.2%) |  |  | 33.0 / 125.0 (26.4%) | 6.0 / 45.0 (13.3%) | 27.0 / 80.0 (33.8%) |  |
| Chronic bronchitis | |  | 91.0 / 265.0 (34.3%) | 52.0 / 136.0 (38.2%) | 39.0 / 129.0 (30.2%) |  |  | 39.0 / 125.0 (31.2%) | 21.0 / 45.0 (46.7%) | 18.0 / 80.0 (22.5%) |  |
| others | |  | 68.0 / 265.0 (25.7%) | 26.0 / 136.0 (19.1%) | 42.0 / 129.0 (32.6%) |  |  | 53.0 / 125.0 (42.4%) | 18.0 / 45.0 (40.0%) | 35.0 / 80.0 (43.8%) |  |
| Missing | |  | 866 | 234 | 632 |  |  | 539 | 79 | 460 |  |
| **Allergic asthma^3^** | | 1,112 / 1,131 |  |  |  | <0.001 | 657 / 664 |  |  |  | 0.090 |
| no | |  | 1,006.0 / 1,112.0 (90.5%) | 309.0 / 363.0 (85.1%) | 697.0 / 749.0 (93.1%) |  |  | 619.0 / 657.0 (94.2%) | 111.0 / 122.0 (91.0%) | 508.0 / 535.0 (95.0%) |  |
| yes | |  | 106.0 / 1,112.0 (9.5%) | 54.0 / 363.0 (14.9%) | 52.0 / 749.0 (6.9%) |  |  | 38.0 / 657.0 (5.8%) | 11.0 / 122.0 (9.0%) | 27.0 / 535.0 (5.0%) |  |
| Missing | |  | 19 | 7 | 12 |  |  | 7 | 2 | 5 |  |
| **Allergic rhinitis^3^** | | 1,116 / 1,131 |  |  |  | 0.26 | 658 / 664 |  |  |  | 0.58 |
| no | |  | 878.0 / 1,116.0 (78.7%) | 280.0 / 365.0 (76.7%) | 598.0 / 751.0 (79.6%) |  |  | 546.0 / 658.0 (83.0%) | 105.0 / 124.0 (84.7%) | 441.0 / 534.0 (82.6%) |  |
| yes | |  | 238.0 / 1,116.0 (21.3%) | 85.0 / 365.0 (23.3%) | 153.0 / 751.0 (20.4%) |  |  | 112.0 / 658.0 (17.0%) | 19.0 / 124.0 (15.3%) | 93.0 / 534.0 (17.4%) |  |
| Missing | |  | 15 | 5 | 10 |  |  | 6 | 0 | 6 |  |
| **Skin disease^3^** | | 279 / 1,131 |  |  |  | 0.81 | 157 / 664 |  |  |  | 0.34 |
| Acne | |  | 43.0 / 279.0 (15.4%) | 17.0 / 91.0 (18.7%) | 26.0 / 188.0 (13.8%) |  |  | 16.0 / 157.0 (10.2%) | 4.0 / 27.0 (14.8%) | 12.0 / 130.0 (9.2%) |  |
| Atopic eczema | |  | 78.0 / 279.0 (28.0%) | 23.0 / 91.0 (25.3%) | 55.0 / 188.0 (29.3%) |  |  | 34.0 / 157.0 (21.7%) | 5.0 / 27.0 (18.5%) | 29.0 / 130.0 (22.3%) |  |
| Light allergy | |  | 10.0 / 279.0 (3.6%) | 3.0 / 91.0 (3.3%) | 7.0 / 188.0 (3.7%) |  |  | 3.0 / 157.0 (1.9%) | 0.0 / 27.0 (0.0%) | 3.0 / 130.0 (2.3%) |  |
| others | |  | 85.0 / 279.0 (30.5%) | 29.0 / 91.0 (31.9%) | 56.0 / 188.0 (29.8%) |  |  | 49.0 / 157.0 (31.2%) | 5.0 / 27.0 (18.5%) | 44.0 / 130.0 (33.8%) |  |
| Psoriasis | |  | 63.0 / 279.0 (22.6%) | 19.0 / 91.0 (20.9%) | 44.0 / 188.0 (23.4%) |  |  | 55.0 / 157.0 (35.0%) | 13.0 / 27.0 (48.1%) | 42.0 / 130.0 (32.3%) |  |
| Missing | |  | 852 | 279 | 573 |  |  | 507 | 97 | 410 |  |
| **IBD^3^** | | 1,117 / 1,131 |  |  |  | 0.005 | 656 / 664 |  |  |  | 0.19 |
| no | |  | 1,060.0 / 1,117.0 (94.9%) | 357.0 / 366.0 (97.5%) | 703.0 / 751.0 (93.6%) |  |  | 610.0 / 656.0 (93.0%) | 111.0 / 123.0 (90.2%) | 499.0 / 533.0 (93.6%) |  |
| yes | |  | 57.0 / 1,117.0 (5.1%) | 9.0 / 366.0 (2.5%) | 48.0 / 751.0 (6.4%) |  |  | 46.0 / 656.0 (7.0%) | 12.0 / 123.0 (9.8%) | 34.0 / 533.0 (6.4%) |  |
| Missing | |  | 14 | 4 | 10 |  |  | 8 | 1 | 7 |  |
| **IBS^3^** | | 1,108 / 1,131 |  |  |  | 0.057 | 654 / 664 |  |  |  | 0.44 |
| no | |  | 1,065.0 / 1,108.0 (96.1%) | 347.0 / 367.0 (94.6%) | 718.0 / 741.0 (96.9%) |  |  | 643.0 / 654.0 (98.3%) | 118.0 / 121.0 (97.5%) | 525.0 / 533.0 (98.5%) |  |
| yes | |  | 43.0 / 1,108.0 (3.9%) | 20.0 / 367.0 (5.4%) | 23.0 / 741.0 (3.1%) |  |  | 11.0 / 654.0 (1.7%) | 3.0 / 121.0 (2.5%) | 8.0 / 533.0 (1.5%) |  |
| Missing | |  | 23 | 3 | 20 |  |  | 10 | 3 | 7 |  |
| **Cancer^3^** | | 1,112 / 1,131 |  |  |  | 0.94 | 656 / 664 |  |  |  | 0.49 |
| no | |  | 1,001.0 / 1,112.0 (90.0%) | 328.0 / 364.0 (90.1%) | 673.0 / 748.0 (90.0%) |  |  | 603.0 / 656.0 (91.9%) | 114.0 / 122.0 (93.4%) | 489.0 / 534.0 (91.6%) |  |
| yes | |  | 111.0 / 1,112.0 (10.0%) | 36.0 / 364.0 (9.9%) | 75.0 / 748.0 (10.0%) |  |  | 53.0 / 656.0 (8.1%) | 8.0 / 122.0 (6.6%) | 45.0 / 534.0 (8.4%) |  |
| Missing | |  | 19 | 6 | 13 |  |  | 8 | 2 | 6 |  |
| **Periodontits^3^** | | 1,111 / 1,131 |  |  |  | 0.46 | 654 / 664 |  |  |  | 0.024 |
| no | |  | 859.0 / 1,111.0 (77.3%) | 272.0 / 358.0 (76.0%) | 587.0 / 753.0 (78.0%) |  |  | 489.0 / 654.0 (74.8%) | 80.0 / 120.0 (66.7%) | 409.0 / 534.0 (76.6%) |  |
| yes | |  | 252.0 / 1,111.0 (22.7%) | 86.0 / 358.0 (24.0%) | 166.0 / 753.0 (22.0%) |  |  | 165.0 / 654.0 (25.2%) | 40.0 / 120.0 (33.3%) | 125.0 / 534.0 (23.4%) |  |
| Missing | |  | 20 | 12 | 8 |  |  | 10 | 4 | 6 |  |
| **Rheumatoid arthritis^3^** | | 1,092 / 1,131 |  |  |  | 0.50 | 643 / 664 |  |  |  | 0.28 |
| no | |  | 990.0 / 1,092.0 (90.7%) | 317.0 / 353.0 (89.8%) | 673.0 / 739.0 (91.1%) |  |  | 603.0 / 643.0 (93.8%) | 117.0 / 122.0 (95.9%) | 486.0 / 521.0 (93.3%) |  |
| yes | |  | 102.0 / 1,092.0 (9.3%) | 36.0 / 353.0 (10.2%) | 66.0 / 739.0 (8.9%) |  |  | 40.0 / 643.0 (6.2%) | 5.0 / 122.0 (4.1%) | 35.0 / 521.0 (6.7%) |  |
| Missing | |  | 39 | 17 | 22 |  |  | 21 | 2 | 19 |  |
| **Regular use of medication^6^** | | 1,119 / 1,131 |  |  |  | <0.001 | 659 / 664 |  |  |  | <0.001 |
| no | |  | 313.0 / 1,119.0 (28.0%) | 52.0 / 365.0 (14.2%) | 261.0 / 754.0 (34.6%) |  |  | 242.0 / 659.0 (36.7%) | 16.0 / 123.0 (13.0%) | 226.0 / 536.0 (42.2%) |  |
| yes | |  | 806.0 / 1,119.0 (72.0%) | 313.0 / 365.0 (85.8%) | 493.0 / 754.0 (65.4%) |  |  | 417.0 / 659.0 (63.3%) | 107.0 / 123.0 (87.0%) | 310.0 / 536.0 (57.8%) |  |
| Missing | |  | 12 | 5 | 7 |  |  | 5 | 1 | 4 |  |
| ^1^Median (IQR), Mean (SD) and Frequencies (N/%) | | | | | | | | | | | |
| ^2^Pearson's Chi-squared test; Wilcoxon rank sum test; Fisher's exact test  ^3^Self-reported  ^4^A fasting blood sugar level from 100 to 125 mg/dL (5.6 to 7.0 mmol/L) is considered as prediabetes.  ^5^Self-reported diabetes mellitus type 2 and diagnosed by high basal glucose levels (a fasting blood sugar level of 126 mg/dL (7.0 mmol/L) or higher indicates diabetes mellitus type 2 diabetes).  ^6^Self-reported and prescription list of the general practitioner. | | | | | | | | | | | |
| Abbreviations: MIG= metabolic inflammation group, ROG= registration office group, BMI= body mass index (BMI class: UW= underweight, NW= normal weight, OW= overweight, OBI= obesity grade I, OBII= obesity grade II, OBIII= obesity grade III), BP= blood pressure, HDL= high density lipoprotein, LDL= low density lipoprotein, CRP= C-reactive protein, IL-6= interleukin 6, IBD= inflammatory bowel disease and IBS= irritable bowel syndrome. | | | | | | | | | | | |
