# Supplementary material for: Cohort profile: the Food Chain Plus (FoCus) cohort
Source: Eur J Epidemiol. 2022 Oct 16;37(10):1087–105. doi: 10.1007/s10654-022-00924-y (PMC9630232; doi:10.1007/s10654-022-00924-y)
Supplement: Supplementary file 2 — Supplementary file2 (DOCX 19 kb) [file 10654_2022_924_MOESM2_ESM.docx]

**Table S2** Activity and nutrition parameters of the FoCus cohort subjects at baseline stratified by sex and type of recruitment

|  | **Females** | | | | | **Males** | | | | |
| --- | --- | --- | --- | --- | --- | --- | --- | --- | --- | --- |
| **Characteristics** | N | Overall, N = 1,051^1^ | MIG, N = 335^1^ | ROG, N = 716^1^ | p-value^2^ | N | Overall, N = 620^1^ | MIG, N = 116^1^ | ROG, N = 504^1^ | p-value^2^ |
| **TV-watching (h/day)** | 1,050 / 1,050 | 2.0 (1.5, 4.0) | 3.0 (2.0, 4.0) | 2.0 (1.5, 3.0) | <0.001 | 620 / 620 | 3.0 (2.0, 4.0) | 4.0 (2.0, 5.0) | 2.0 (1.5, 3.0) | <0.001 |
| Missing |  | 0 | 0 | 0 |  |  | 0 | 0 | 0 |  |
| **Daily activity (min./week)** | 1,050 / 1,050 | 1,050.0 (615.0, 1,631.2) | 967.5 (570.0, 1,582.5) | 1,117.5 (645.0, 1,650.0) | 0.026 | 620 / 620 | 720.0 (470.6, 1,179.4) | 660.0 (431.2, 993.8) | 750.0 (480.0, 1,200.0) | 0.051 |
| Missing |  | 0 | 0 | 0 |  |  | 0 | 0 | 0 |  |
| **Sports activity (min./week)** | 1,050 / 1,050 | 183.8 (60.0, 360.0) | 97.5 (0.0, 240.0) | 240.0 (97.5, 390.0) | <0.001 | 620 / 620 | 240.0 (67.5, 420.0) | 90.0 (0.0, 300.0) | 251.2 (105.0, 450.0) | <0.001 |
| Missing |  | 0 | 0 | 0 |  |  | 0 | 0 | 0 |  |
| **Sleep (h/night)** | 1,050 / 1,050 | 7.0 (6.0, 8.0) | 7.0 (6.0, 8.0) | 7.0 (7.0, 8.0) | 0.059 | 620 / 620 | 7.0 (6.0, 8.0) | 7.0 (6.0, 8.0) | 7.0 (6.0, 8.0) | 0.022 |
| Missing |  | 0 | 0 | 0 |  |  | 0 | 0 | 0 |  |
| **Energy (kJ/day)** | 1,050 / 1,050 | 8,061.9 (6,724.1, 9,626.6) | 8,194.7 (6,675.5, 9,962.3) | 7,985.0 (6,739.4, 9,421.5) | 0.14 | 620 / 620 | 10,326.2 (8,462.8, 12,736.7) | 10,471.8 (8,008.9, 13,830.1) | 10,307.6 (8,546.4, 12,502.4) | 0.76 |
| Missing |  | 0 | 0 | 0 |  |  | 0 | 0 | 0 |  |
| **Carbohydrates (% Energy)** | 1,050 / 1,050 | 42.9 (39.6, 46.7) | 43.2 (39.5, 47.8) | 42.7 (39.6, 46.4) | 0.15 | 620 / 620 | 39.3 (35.7, 42.8) | 40.3 (36.4, 43.4) | 39.2 (35.6, 42.6) | 0.30 |
| Missing |  | 0 | 0 | 0 |  |  | 0 | 0 | 0 |  |
| **Proteins (% Energy)** | 1,050 / 1,050 | 14.4 (13.2, 15.7) | 15.1 (13.9, 16.5) | 14.1 (12.9, 15.3) | <0.001 | 620 / 620 | 15.1 (13.6, 16.5) | 15.9 (14.6, 17.6) | 14.9 (13.5, 16.4) | <0.001 |
|  |  |  |  |  |  |  |  |  |  |  |
| **Fats (% Energy)** | 1,050 / 1,050 | 38.9 (35.6, 41.8) | 39.5 (35.2, 42.4) | 38.8 (35.7, 41.7) | 0.78 | 620 / 620 | 40.8 (37.1, 44.1) | 41.5 (36.9, 44.8) | 40.8 (37.2, 43.9) | 0.46 |
| Missing |  | 0 | 0 | 0 |  |  | 0 | 0 | 0 |  |
| **Fibres (g/day)** | 1,050 / 1,050 | 22.4 (19.7, 25.9) | 22.2 (19.2, 26.2) | 22.5 (20.0, 25.9) | 0.16 | 620 / 620 | 20.3 (17.6, 23.9) | 20.8 (17.7, 24.1) | 20.2 (17.5, 23.8) | 0.72 |
| Missing |  | 0 | 0 | 0 |  |  | 0 | 0 | 0 |  |
| **Organic acids (g/day)** | 1,050 / 1,050 | 8.0 (6.7, 9.4) | 8.1 (6.7, 9.9) | 8.0 (6.7, 9.4) | 0.14 | 620 / 620 | 6.3 (4.4, 7.9) | 6.7 (4.6, 8.6) | 6.1 (4.4, 7.8) | 0.076 |
| Missing |  | 0 | 0 | 0 |  |  | 0 | 0 | 0 |  |
| **Minerals (g/day)** | 1,050 / 1,050 | 16.5 (15.5, 17.7) | 16.9 (15.5, 18.1) | 16.4 (15.5, 17.4) | 0.001 | 620 / 620 | 17.1 (16.0, 18.4) | 17.7 (16.3, 18.9) | 17.0 (15.9, 18.3) | 0.017 |
| Missing |  | 0 | 0 | 0 |  |  | 0 | 0 | 0 |  |
| **Alcohol intake (g/day)** | 1,050 / 1,050 | 1.5 (0.5, 3.7) | 0.6 (0.3, 1.6) | 2.2 (0.9, 4.4) | <0.001 | 620 / 620 | 2.9 (1.0, 5.4) | 1.3 (0.4, 2.8) | 3.4 (1.3, 5.8) | <0.001 |
| Missing |  | 0 | 0 | 0 |  |  | 0 | 0 | 0 |  |
| **Salt intake (g/day)** | 1,050 / 1,050 | 5.5 (5.0, 6.0) | 5.7 (5.2, 6.2) | 5.4 (5.0, 5.8) | <0.001 | 620 / 620 | 5.9 (5.3, 6.6) | 6.3 (5.5, 7.2) | 5.8 (5.2, 6.5) | <0.001 |
| Missing |  | 0 | 0 | 0 |  |  | 0 | 0 | 0 |  |
| ^1^Median (IQR) | | | | | | | | | | |
| ^2^Wilcoxon rank sum test | | | | | | | | | | |
| Abbreviations: MIG= metabolic inflammation group, ROG= registration office group, h= hours, min.= minutes. | | | | | | | | | | |
