# Supplementary material for: Cohort profile: the Food Chain Plus (FoCus) cohort
Source: Eur J Epidemiol. 2022 Oct 16;37(10):1087–105. doi: 10.1007/s10654-022-00924-y (PMC9630232; doi:10.1007/s10654-022-00924-y)
Supplement: Supplementary file 3 — Supplementary file3 (DOCX 41 kb) [file 10654_2022_924_MOESM3_ESM.docx]

**Table S3** Demographic, clinical and laboratory characteristics of the FoCus cohort subjects at baseline stratified by type of recruitment and responder status

|  | | | **MIG** | | | | **ROG** | | | | |
| --- | --- | --- | --- | --- | --- | --- | --- | --- | --- | --- | --- |
| **Characteristics** | **N** | **Overall, N = 494^1^** | | **Nonresponder,**  **N = 295^1^** | **Responder*,**  **N = 199^1^** | **p-value^2^** | **N** | **Overall, N = 1,301^1^** | **Nonresponder, N = 682^1^** | **Responder,**  **N = 619^1^** | **p-value^2^** |
| **Sex** | 494 / 494 |  | |  |  | 0.66 | 1,301 / 1,301 |  |  |  | 0.26 |
| Females |  | 370.0 / 494.0 (74.9%) | | 223.0 / 295.0 (75.6%) | 147.0 / 199.0 (73.9%) |  |  | 761.0 / 1,301.0 (58.5%) | 389.0 / 682.0 (57.0%) | 372.0 / 619.0 (60.1%) |  |
| Males |  | 124.0 / 494.0 (25.1%) | | 72.0 / 295.0 (24.4%) | 52.0 / 199.0 (26.1%) |  |  | 540.0 / 1,301.0 (41.5%) | 293.0 / 682.0 (43.0%) | 247.0 / 619.0 (39.9%) |  |
| Missing |  | 0 | | 0 | 0 |  |  | 0 | 0 | 0 |  |
| **Age (years)** | 494 / 494 | 48.0 (40.0, 57.0) | | 47.0 (38.0, 55.0) | 51.0 (43.0, 60.0) | <0.001 | 1,301 / 1,301 | 54.0 (44.0, 65.0) | 51.0 (40.0, 63.0) | 57.0 (48.0, 67.0) | <0.001 |
| Missing |  | 0 | | 0 | 0 |  |  | 0 | 0 | 0 |  |
| **Height (cm)** | 494 / 494 | 170.0 (164.6, 177.9) | | 170.0 (165.0, 177.8) | 170.0 (164.0, 177.5) | 0.81 | 1,301 / 1,301 | 172.0 (167.0, 180.0) | 172.5 (167.0, 179.5) | 172.0 (167.0, 180.0) | 0.82 |
| Missing |  | 0 | | 0 | 0 |  |  | 0 | 0 | 0 |  |
| **Weight (kg)** | 494 / 494 | 123.8 (103.3, 144.2) | | 124.2 (103.4, 144.6) | 121.3 (102.4, 142.7) | 0.45 | 1,301 / 1,301 | 76.3 (65.8, 88.7) | 76.9 (65.5, 90.4) | 76.0 (66.0, 87.1) | 0.33 |
| Missing |  | 0 | | 0 | 0 |  |  | 0 | 0 | 0 |  |
| **BMI (kg/m²)** | 494 / 494 | 42.8 (36.7, 48.8) | | 42.7 (36.9, 48.9) | 42.8 (36.0, 48.5) | 0.49 | 1,301 / 1,301 | 25.4 (22.7, 28.6) | 25.7 (22.5, 29.3) | 25.2 (22.8, 28.1) | 0.37 |
| Range |  | 19.0, 72.7 | | 19.0, 67.1 | 22.8, 72.7 |  |  | 14.5, 83.2 | 14.5, 83.2 | 15.7, 49.6 |  |
| Missing |  | 0 | | 0 | 0 |  |  | 0 | 0 | 0 |  |
| **BMI class** | 494 / 494 |  | |  |  | 0.36 | 1,301 / 1,301 |  |  |  | 0.36 |
| UW (< 18.5 kg/m²) |  |  | |  |  |  |  | 24.0 / 1,301.0 (1.8%) | 14.0 / 682.0 (2.1%) | 10.0 / 619.0 (1.6%) |  |
| NW (18.5 to 24.9 kg/m²) |  | 5.0 / 494.0 (1.0%) | | 3.0 / 295.0 (1.0%) | 2.0 / 199.0 (1.0%) |  |  | 565.0 / 1,301.0 (43.4%) | 286.0 / 682.0 (41.9%) | 279.0 / 619.0 (45.1%) |  |
| OW (25.0 to 29.9 kg/m²) |  | 25.0 / 494.0 (5.1%) | | 10.0 / 295.0 (3.4%) | 15.0 / 199.0 (7.5%) |  |  | 458.0 / 1,301.0 (35.2%) | 233.0 / 682.0 (34.2%) | 225.0 / 619.0 (36.3%) |  |
| OBI (30.0 to 34.9 kg/m²) |  | 72.0 / 494.0 (14.6%) | | 45.0 / 295.0 (15.3%) | 27.0 / 199.0 (13.6%) |  |  | 168.0 / 1,301.0 (12.9%) | 99.0 / 682.0 (14.5%) | 69.0 / 619.0 (11.1%) |  |
| OBII (35.0 to 39.9 kg/m²) |  | 83.0 / 494.0 (16.8%) | | 51.0 / 295.0 (17.3%) | 32.0 / 199.0 (16.1%) |  |  | 57.0 / 1,301.0 (4.4%) | 33.0 / 682.0 (4.8%) | 24.0 / 619.0 (3.9%) |  |
| OBIII (≥ 40.0 kg/m²) |  | 309.0 / 494.0 (62.6%) | | 186.0 / 295.0 (63.1%) | 123.0 / 199.0 (61.8%) |  |  | 29.0 / 1,301.0 (2.2%) | 17.0 / 682.0 (2.5%) | 12.0 / 619.0 (1.9%) |  |
| Missing |  | 0 | | 0 | 0 |  |  | 0 | 0 | 0 |  |
| **Hip-circumference (cm)** | 352 / 494 | 129.0 (120.0, 139.0) | | 129.0 (120.0, 139.0) | 129.0 (119.0, 141.0) | 0.69 | 1,287 / 1,301 | 105.0 (100.0, 111.2) | 106.0 (100.0, 112.5) | 105.0 (101.0, 110.0) | 0.33 |
| Missing |  | 142 | | 90 | 52 |  |  | 14 | 9 | 5 |  |
| **Waist-circumference (cm)** | 370 / 494 | 123.0 (112.0, 134.0) | | 122.0 (112.0, 134.0) | 124.0 (113.0, 133.5) | 0.83 | 1,290 / 1,301 | 92.0 (81.0, 103.0) | 92.0 (80.0, 104.0) | 92.0 (82.0, 102.0) | 0.91 |
| Missing |  | 124 | | 80 | 44 |  |  | 11 | 8 | 3 |  |
| **BP systolic (mmHg)** | 494 / 494 | 133.7 (10.9) | | 133.9 (11.0) | 133.6 (10.6) | 0.43 | 1,301 / 1,301 | 127.3 (12.0) | 126.4 (11.7) | 128.3 (12.2) | 0.006 |
| Missing |  | 0 | | 0 | 0 |  |  | 0 | 0 | 0 |  |
| **BP diastolic (mmHg)** | 494 / 494 | 83.0 (6.7) | | 83.6 (6.6) | 82.1 (6.8) | 0.016 | 1,301 / 1,301 | 79.4 (6.4) | 79.3 (6.4) | 79.5 (6.5) | 0.66 |
| Missing |  | 0 | | 0 | 0 |  |  | 0 | 0 | 0 |  |
| **Triglycerides (mg/dL)** | 491 / 494 | 140.0 (102.5, 194.5) | | 136.0 (103.0, 189.8) | 143.0 (100.0, 199.0) | 0.82 | 1,299 / 1,301 | 98.0 (70.0, 139.0) | 101.0 (71.0, 144.0) | 94.0 (70.0, 132.0) | 0.070 |
| Missing |  | 3 | | 1 | 2 |  |  | 2 | 2 | 0 |  |
| **Cholesterol total (mmol/L)** | 359 / 494 | 4.5 (4.0, 5.1) | | 4.5 (4.0, 5.1) | 4.4 (4.0, 5.1) | 0.77 | 608 / 1,301 | 4.6 (4.0, 5.2) | 4.6 (4.0, 5.2) | 4.6 (4.0, 5.2) | 0.42 |
| Missing |  | 135 | | 79 | 56 |  |  | 693 | 344 | 349 |  |
| **LDL-cholesterol (mmol/L)** | 0 / 494 | NA (NA, NA) | | NA (NA, NA) | NA (NA, NA) |  | 123 / 1,301 | 3.1 (2.6, 3.7) | 3.0 (2.6, 3.6) | 3.1 (2.5, 3.8) | 0.54 |
| Missing |  | 494 | | 295 | 199 |  |  | 1,178 | 614 | 564 |  |
| **HDL-cholesterol (mmol/L)** | 0 / 494 | NA (NA, NA) | | NA (NA, NA) | NA (NA, NA) |  | 123 / 1,301 | 1.5 (1.3, 1.9) | 1.6 (1.3, 1.8) | 1.4 (1.2, 1.9) | 0.85 |
| Missing |  | 494 | | 295 | 199 |  |  | 1,178 | 614 | 564 |  |
| **Lipoprotein a (mg/L)** | 231 / 494 | 264.0 (154.5, 492.0) | | 278.0 (155.5, 499.5) | 255.0 (151.0, 480.0) | 0.89 | 444 / 1,301 | 249.5 (136.0, 485.0) | 245.0 (136.0, 478.5) | 252.0 (137.2, 485.0) | 0.81 |
| Missing |  | 263 | | 157 | 106 |  |  | 857 | 448 | 409 |  |
| **Glucose (mg/dL)** | 491 / 494 | 101.0 (91.0, 117.0) | | 101.0 (90.0, 117.8) | 102.0 (92.0, 117.0) | 0.40 | 1,298 / 1,301 | 93.0 (88.0, 101.0) | 93.0 (87.0, 101.0) | 94.0 (88.0, 101.0) | 0.37 |
| Missing |  | 3 | | 1 | 2 |  |  | 3 | 2 | 1 |  |
| **Insulin (mU/L)** | 487 / 494 | 19.2 (11.8, 34.0) | | 19.3 (11.8, 33.0) | 18.6 (11.5, 35.1) | 0.89 | 1,291 / 1,301 | 8.6 (6.0, 12.9) | 9.2 (6.1, 14.1) | 8.1 (5.8, 11.9) | 0.002 |
| Missing |  | 7 | | 2 | 5 |  |  | 10 | 5 | 5 |  |
| **HOMA-IR** | 489 / 494 | 4.8 (2.7, 9.4) | | 4.8 (2.8, 9.2) | 4.8 (2.7, 9.8) | 0.72 | 1,296 / 1,301 | 2.0 (1.3, 3.1) | 2.1 (1.3, 3.4) | 1.9 (1.3, 2.9) | 0.008 |
| Missing |  | 5 | | 1 | 4 |  |  | 5 | 3 | 2 |  |
| **CRP (mg/L)** | 463 / 494 | 6.4 (3.4, 11.1) | | 6.0 (3.3, 10.9) | 7.0 (3.6, 11.6) | 0.47 | 746 / 1,301 | 2.3 (1.4, 3.9) | 2.5 (1.5, 4.3) | 2.1 (1.3, 3.5) | 0.003 |
| Missing |  | 31 | | 13 | 18 |  |  | 555 | 305 | 250 |  |
| **IL-6 (pg/mL)** | 457 / 494 | 4.9 (3.5, 6.8) | | 5.1 (3.6, 7.0) | 4.7 (3.5, 6.7) | 0.37 | 996 / 1,301 | 3.4 (2.4, 4.8) | 3.4 (2.4, 4.7) | 3.4 (2.4, 4.9) | >0.99 |
| Missing |  | 37 | | 26 | 11 |  |  | 305 | 160 | 145 |  |
| **Smoking habits** | 470 / 494 |  | |  |  | 0.21 | 1,274 / 1,301 |  |  |  | <0.001 |
| Never smoking |  | 143.0 / 470.0 (30.4%) | | 77.0 / 279.0 (27.6%) | 66.0 / 191.0 (34.6%) |  |  | 484.0 / 1,274.0 (38.0%) | 258.0 / 668.0 (38.6%) | 226.0 / 606.0 (37.3%) |  |
| Previous smoking |  | 194.0 / 470.0 (41.3%) | | 114.0 / 279.0 (40.9%) | 80.0 / 191.0 (41.9%) |  |  | 479.0 / 1,274.0 (37.6%) | 226.0 / 668.0 (33.8%) | 253.0 / 606.0 (41.7%) |  |
| Less than 3 months |  | 35.0 / 470.0 (7.4%) | | 24.0 / 279.0 (8.6%) | 11.0 / 191.0 (5.8%) |  |  | 89.0 / 1,274.0 (7.0%) | 37.0 / 668.0 (5.5%) | 52.0 / 606.0 (8.6%) |  |
| Smoking |  | 98.0 / 470.0 (20.9%) | | 64.0 / 279.0 (22.9%) | 34.0 / 191.0 (17.8%) |  |  | 222.0 / 1,274.0 (17.4%) | 147.0 / 668.0 (22.0%) | 75.0 / 606.0 (12.4%) |  |
| Missing |  | 24 | | 16 | 8 |  |  | 27 | 14 | 13 |  |
| **School education** | 487 / 494 |  | |  |  | 0.20 | 1,297 / 1,301 |  |  |  | 0.73 |
| University qualification |  | 84.0 / 487.0 (17.2%) | | 47.0 / 291.0 (16.2%) | 37.0 / 196.0 (18.9%) |  |  | 459.0 / 1,297.0 (35.4%) | 233.0 / 680.0 (34.3%) | 226.0 / 617.0 (36.6%) |  |
| Technical college qualification |  | 42.0 / 487.0 (8.6%) | | 21.0 / 291.0 (7.2%) | 21.0 / 196.0 (10.7%) |  |  | 154.0 / 1,297.0 (11.9%) | 82.0 / 680.0 (12.1%) | 72.0 / 617.0 (11.7%) |  |
| Middle school |  | 184.0 / 487.0 (37.8%) | | 119.0 / 291.0 (40.9%) | 65.0 / 196.0 (33.2%) |  |  | 429.0 / 1,297.0 (33.1%) | 227.0 / 680.0 (33.4%) | 202.0 / 617.0 (32.7%) |  |
| Secondary school |  | 167.0 / 487.0 (34.3%) | | 96.0 / 291.0 (33.0%) | 71.0 / 196.0 (36.2%) |  |  | 250.0 / 1,297.0 (19.3%) | 134.0 / 680.0 (19.7%) | 116.0 / 617.0 (18.8%) |  |
| No degree |  | 10.0 / 487.0 (2.1%) | | 8.0 / 291.0 (2.7%) | 2.0 / 196.0 (1.0%) |  |  | 5.0 / 1,297.0 (0.4%) | 4.0 / 680.0 (0.6%) | 1.0 / 617.0 (0.2%) |  |
| Missing |  | 7 | | 4 | 3 |  |  | 4 | 2 | 2 |  |
| **Employment** | 491 / 494 |  | |  |  | 0.057 | 1,292 / 1,301 |  |  |  | <0.001 |
| Full time |  | 147.0 / 491.0 (29.9%) | | 89.0 / 294.0 (30.3%) | 58.0 / 197.0 (29.4%) |  |  | 456.0 / 1,292.0 (35.3%) | 247.0 / 676.0 (36.5%) | 209.0 / 616.0 (33.9%) |  |
| Part time |  | 86.0 / 491.0 (17.5%) | | 55.0 / 294.0 (18.7%) | 31.0 / 197.0 (15.7%) |  |  | 268.0 / 1,292.0 (20.7%) | 149.0 / 676.0 (22.0%) | 119.0 / 616.0 (19.3%) |  |
| Unemployed |  | 68.0 / 491.0 (13.8%) | | 47.0 / 294.0 (16.0%) | 21.0 / 197.0 (10.7%) |  |  | 40.0 / 1,292.0 (3.1%) | 31.0 / 676.0 (4.6%) | 9.0 / 616.0 (1.5%) |  |
| Retired |  | 122.0 / 491.0 (24.8%) | | 60.0 / 294.0 (20.4%) | 62.0 / 197.0 (31.5%) |  |  | 402.0 / 1,292.0 (31.1%) | 178.0 / 676.0 (26.3%) | 224.0 / 616.0 (36.4%) |  |
| other |  | 68.0 / 491.0 (13.8%) | | 43.0 / 294.0 (14.6%) | 25.0 / 197.0 (12.7%) |  |  | 126.0 / 1,292.0 (9.8%) | 71.0 / 676.0 (10.5%) | 55.0 / 616.0 (8.9%) |  |
| Missing |  | 3 | | 1 | 2 |  |  | 9 | 6 | 3 |  |
| **Content of life** | 479 / 494 |  | |  |  | 0.049 | 1,285 / 1,301 |  |  |  | <0.001 |
| Very content |  | 39.0 / 479.0 (8.1%) | | 18.0 / 285.0 (6.3%) | 21.0 / 194.0 (10.8%) |  |  | 364.0 / 1,285.0 (28.3%) | 162.0 / 673.0 (24.1%) | 202.0 / 612.0 (33.0%) |  |
| Content |  | 193.0 / 479.0 (40.3%) | | 110.0 / 285.0 (38.6%) | 83.0 / 194.0 (42.8%) |  |  | 766.0 / 1,285.0 (59.6%) | 411.0 / 673.0 (61.1%) | 355.0 / 612.0 (58.0%) |  |
| Not so content |  | 185.0 / 479.0 (38.6%) | | 112.0 / 285.0 (39.3%) | 73.0 / 194.0 (37.6%) |  |  | 136.0 / 1,285.0 (10.6%) | 87.0 / 673.0 (12.9%) | 49.0 / 612.0 (8.0%) |  |
| Not at all content |  | 62.0 / 479.0 (12.9%) | | 45.0 / 285.0 (15.8%) | 17.0 / 194.0 (8.8%) |  |  | 19.0 / 1,285.0 (1.5%) | 13.0 / 673.0 (1.9%) | 6.0 / 612.0 (1.0%) |  |
| Missing |  | 15 | | 10 | 5 |  |  | 16 | 9 | 7 |  |
| **Household type** | 492 / 494 |  | |  |  | 0.044 | 1,288 / 1,301 |  |  |  | 0.009 |
| Living with partner |  | 318.0 / 492.0 (64.6%) | | 178.0 / 295.0 (60.3%) | 140.0 / 197.0 (71.1%) |  |  | 996.0 / 1,288.0 (77.3%) | 504.0 / 672.0 (75.0%) | 492.0 / 616.0 (79.9%) |  |
| Living alone |  | 130.0 / 492.0 (26.4%) | | 89.0 / 295.0 (30.2%) | 41.0 / 197.0 (20.8%) |  |  | 226.0 / 1,288.0 (17.5%) | 122.0 / 672.0 (18.2%) | 104.0 / 616.0 (16.9%) |  |
| Other types |  | 44.0 / 492.0 (8.9%) | | 28.0 / 295.0 (9.5%) | 16.0 / 197.0 (8.1%) |  |  | 66.0 / 1,288.0 (5.1%) | 46.0 / 672.0 (6.8%) | 20.0 / 616.0 (3.2%) |  |
| Missing |  | 2 | | 0 | 2 |  |  | 13 | 10 | 3 |  |
| **Children** | 489 / 494 |  | |  |  | 0.25 | 1,296 / 1,301 |  |  |  | 0.003 |
| no |  | 181.0 / 489.0 (37.0%) | | 114.0 / 291.0 (39.2%) | 67.0 / 198.0 (33.8%) |  |  | 369.0 / 1,296.0 (28.5%) | 216.0 / 677.0 (31.9%) | 153.0 / 619.0 (24.7%) |  |
| yes |  | 308.0 / 489.0 (63.0%) | | 177.0 / 291.0 (60.8%) | 131.0 / 198.0 (66.2%) |  |  | 925.0 / 1,296.0 (71.4%) | 459.0 / 677.0 (67.8%) | 466.0 / 619.0 (75.3%) |  |
| unknown |  | 0.0 / 489.0 (0.0%) | | 0.0 / 291.0 (0.0%) | 0.0 / 198.0 (0.0%) |  |  | 2.0 / 1,296.0 (0.2%) | 2.0 / 677.0 (0.3%) | 0.0 / 619.0 (0.0%) |  |
| Missing |  | 5 | | 4 | 1 |  |  | 5 | 5 | 0 |  |
| **Diabetes^3^** | 493 / 494 |  | |  |  | 0.28 | 1,298 / 1,301 |  |  |  | 0.65 |
| Normal |  | 197.0 / 493.0 (40.0%) | | 124.0 / 295.0 (42.0%) | 73.0 / 198.0 (36.9%) |  |  | 889.0 / 1,298.0 (68.5%) | 460.0 / 680.0 (67.6%) | 429.0 / 618.0 (69.4%) |  |
| Prediabetes^4^ |  | 124.0 / 493.0 (25.2%) | | 76.0 / 295.0 (25.8%) | 48.0 / 198.0 (24.2%) |  |  | 295.0 / 1,298.0 (22.7%) | 153.0 / 680.0 (22.5%) | 142.0 / 618.0 (23.0%) |  |
| T1DM |  | 2.0 / 493.0 (0.4%) | | 2.0 / 295.0 (0.7%) | 0.0 / 198.0 (0.0%) |  |  | 6.0 / 1,298.0 (0.5%) | 4.0 / 680.0 (0.6%) | 2.0 / 618.0 (0.3%) |  |
| T2DM^5^ |  | 157.0 / 493.0 (31.8%) | | 84.0 / 295.0 (28.5%) | 73.0 / 198.0 (36.9%) |  |  | 96.0 / 1,298.0 (7.4%) | 55.0 / 680.0 (8.1%) | 41.0 / 618.0 (6.6%) |  |
| other |  | 13.0 / 493.0 (2.6%) | | 9.0 / 295.0 (3.1%) | 4.0 / 198.0 (2.0%) |  |  | 12.0 / 1,298.0 (0.9%) | 8.0 / 680.0 (1.2%) | 4.0 / 618.0 (0.6%) |  |
| Missing |  | 1 | | 0 | 1 |  |  | 3 | 2 | 1 |  |
| **Hypertension^3^** | 485 / 494 |  | |  |  | 0.19 | 1,289 / 1,301 |  |  |  | <0.001 |
| no |  | 159.0 / 485.0 (32.8%) | | 101.0 / 288.0 (35.1%) | 58.0 / 197.0 (29.4%) |  |  | 864.0 / 1,289.0 (67.0%) | 486.0 / 677.0 (71.8%) | 378.0 / 612.0 (61.8%) |  |
| yes |  | 326.0 / 485.0 (67.2%) | | 187.0 / 288.0 (64.9%) | 139.0 / 197.0 (70.6%) |  |  | 425.0 / 1,289.0 (33.0%) | 191.0 / 677.0 (28.2%) | 234.0 / 612.0 (38.2%) |  |
| Missing |  | 9 | | 7 | 2 |  |  | 12 | 5 | 7 |  |
| **Dyslipidemia^3^** | 480 / 494 |  | |  |  | 0.065 | 1,275 / 1,301 |  |  |  | 0.011 |
| no |  | 312.0 / 480.0 (65.0%) | | 196.0 / 287.0 (68.3%) | 116.0 / 193.0 (60.1%) |  |  | 922.0 / 1,275.0 (72.3%) | 504.0 / 669.0 (75.3%) | 418.0 / 606.0 (69.0%) |  |
| yes |  | 168.0 / 480.0 (35.0%) | | 91.0 / 287.0 (31.7%) | 77.0 / 193.0 (39.9%) |  |  | 353.0 / 1,275.0 (27.7%) | 165.0 / 669.0 (24.7%) | 188.0 / 606.0 (31.0%) |  |
| Missing |  | 14 | | 8 | 6 |  |  | 26 | 13 | 13 |  |
| **Myocardial infarction^3^** | 487 / 494 |  | |  |  | 0.77 | 1,294 / 1,301 |  |  |  | 0.54 |
| no |  | 471.0 / 487.0 (96.7%) | | 282.0 / 291.0 (96.9%) | 189.0 / 196.0 (96.4%) |  |  | 1,256.0 / 1,294.0 (97.1%) | 659.0 / 677.0 (97.3%) | 597.0 / 617.0 (96.8%) |  |
| yes |  | 16.0 / 487.0 (3.3%) | | 9.0 / 291.0 (3.1%) | 7.0 / 196.0 (3.6%) |  |  | 38.0 / 1,294.0 (2.9%) | 18.0 / 677.0 (2.7%) | 20.0 / 617.0 (3.2%) |  |
| Missing |  | 7 | | 4 | 3 |  |  | 7 | 5 | 2 |  |
| **Cardiac failure^3^** | 481 / 494 |  | |  |  | 0.95 | 1,280 / 1,301 |  |  |  | 0.29 |
| no |  | 450.0 / 481.0 (93.6%) | | 273.0 / 292.0 (93.5%) | 177.0 / 189.0 (93.7%) |  |  | 1,251.0 / 1,280.0 (97.7%) | 653.0 / 671.0 (97.3%) | 598.0 / 609.0 (98.2%) |  |
| yes |  | 31.0 / 481.0 (6.4%) | | 19.0 / 292.0 (6.5%) | 12.0 / 189.0 (6.3%) |  |  | 29.0 / 1,280.0 (2.3%) | 18.0 / 671.0 (2.7%) | 11.0 / 609.0 (1.8%) |  |
| Missing |  | 13 | | 3 | 10 |  |  | 21 | 11 | 10 |  |
| **Liver disease^3^** | 482 / 494 |  | |  |  | 0.078 | 1,291 / 1,301 |  |  |  | 0.20 |
| no |  | 434.0 / 482.0 (90.0%) | | 265.0 / 288.0 (92.0%) | 169.0 / 194.0 (87.1%) |  |  | 1,230.0 / 1,291.0 (95.3%) | 648.0 / 675.0 (96.0%) | 582.0 / 616.0 (94.5%) |  |
| yes |  | 48.0 / 482.0 (10.0%) | | 23.0 / 288.0 (8.0%) | 25.0 / 194.0 (12.9%) |  |  | 61.0 / 1,291.0 (4.7%) | 27.0 / 675.0 (4.0%) | 34.0 / 616.0 (5.5%) |  |
| Missing |  | 12 | | 7 | 5 |  |  | 10 | 7 | 3 |  |
| **Neurological disease^3^** | 479 / 494 |  | |  |  | 0.51 | 1,265 / 1,301 |  |  |  | 0.72 |
| no |  | 377.0 / 479.0 (78.7%) | | 228.0 / 286.0 (79.7%) | 149.0 / 193.0 (77.2%) |  |  | 1,131.0 / 1,265.0 (89.4%) | 589.0 / 661.0 (89.1%) | 542.0 / 604.0 (89.7%) |  |
| yes |  | 102.0 / 479.0 (21.3%) | | 58.0 / 286.0 (20.3%) | 44.0 / 193.0 (22.8%) |  |  | 134.0 / 1,265.0 (10.6%) | 72.0 / 661.0 (10.9%) | 62.0 / 604.0 (10.3%) |  |
| Missing |  | 15 | | 9 | 6 |  |  | 36 | 21 | 15 |  |
| **Stroke^3^** | 492 / 494 |  | |  |  | 0.72 | 1,290 / 1,301 |  |  |  | 0.31 |
| no |  | 484.0 / 492.0 (98.4%) | | 291.0 / 295.0 (98.6%) | 193.0 / 197.0 (98.0%) |  |  | 1,266.0 / 1,290.0 (98.1%) | 660.0 / 675.0 (97.8%) | 606.0 / 615.0 (98.5%) |  |
| yes |  | 8.0 / 492.0 (1.6%) | | 4.0 / 295.0 (1.4%) | 4.0 / 197.0 (2.0%) |  |  | 24.0 / 1,290.0 (1.9%) | 15.0 / 675.0 (2.2%) | 9.0 / 615.0 (1.5%) |  |
| Missing |  | 2 | | 0 | 2 |  |  | 11 | 7 | 4 |  |
| **Respiratory disease^3^** | 181 / 494 |  | |  |  | 0.44 | 209 / 1,301 |  |  |  | 0.040 |
| Asthma |  | 64.0 / 181.0 (35.4%) | | 36.0 / 109.0 (33.0%) | 28.0 / 72.0 (38.9%) |  |  | 75.0 / 209.0 (35.9%) | 38.0 / 108.0 (35.2%) | 37.0 / 101.0 (36.6%) |  |
| Chronic bronchitis |  | 73.0 / 181.0 (40.3%) | | 43.0 / 109.0 (39.4%) | 30.0 / 72.0 (41.7%) |  |  | 57.0 / 209.0 (27.3%) | 37.0 / 108.0 (34.3%) | 20.0 / 101.0 (19.8%) |  |
| others |  | 44.0 / 181.0 (24.3%) | | 30.0 / 109.0 (27.5%) | 14.0 / 72.0 (19.4%) |  |  | 77.0 / 209.0 (36.8%) | 33.0 / 108.0 (30.6%) | 44.0 / 101.0 (43.6%) |  |
| Missing |  | 313 | | 186 | 127 |  |  | 1,092 | 574 | 518 |  |
| **Allergic asthma^3^** | 485 / 494 |  | |  |  | >0.99 | 1,284 / 1,301 |  |  |  | 0.75 |
| no |  | 420.0 / 485.0 (86.6%) | | 252.0 / 291.0 (86.6%) | 168.0 / 194.0 (86.6%) |  |  | 1,205.0 / 1,284.0 (93.8%) | 632.0 / 672.0 (94.0%) | 573.0 / 612.0 (93.6%) |  |
| yes |  | 65.0 / 485.0 (13.4%) | | 39.0 / 291.0 (13.4%) | 26.0 / 194.0 (13.4%) |  |  | 79.0 / 1,284.0 (6.2%) | 40.0 / 672.0 (6.0%) | 39.0 / 612.0 (6.4%) |  |
| Missing |  | 9 | | 4 | 5 |  |  | 17 | 10 | 7 |  |
| **Allergic rhinitis^3^** | 489 / 494 |  | |  |  | 0.35 | 1,285 / 1,301 |  |  |  | 0.65 |
| no |  | 385.0 / 489.0 (78.7%) | | 225.0 / 291.0 (77.3%) | 160.0 / 198.0 (80.8%) |  |  | 1,039.0 / 1,285.0 (80.9%) | 549.0 / 675.0 (81.3%) | 490.0 / 610.0 (80.3%) |  |
| yes |  | 104.0 / 489.0 (21.3%) | | 66.0 / 291.0 (22.7%) | 38.0 / 198.0 (19.2%) |  |  | 246.0 / 1,285.0 (19.1%) | 126.0 / 675.0 (18.7%) | 120.0 / 610.0 (19.7%) |  |
| Missing |  | 5 | | 4 | 1 |  |  | 16 | 7 | 9 |  |
| **Skin disease^3^** | 118 / 494 |  | |  |  | 0.47 | 318 / 1,301 |  |  |  | 0.59 |
| Acne |  | 21.0 / 118.0 (17.8%) | | 10.0 / 75.0 (13.3%) | 11.0 / 43.0 (25.6%) |  |  | 38.0 / 318.0 (11.9%) | 21.0 / 167.0 (12.6%) | 17.0 / 151.0 (11.3%) |  |
| Atopic eczema |  | 28.0 / 118.0 (23.7%) | | 20.0 / 75.0 (26.7%) | 8.0 / 43.0 (18.6%) |  |  | 84.0 / 318.0 (26.4%) | 50.0 / 167.0 (29.9%) | 34.0 / 151.0 (22.5%) |  |
| Light allergy |  | 3.0 / 118.0 (2.5%) | | 2.0 / 75.0 (2.7%) | 1.0 / 43.0 (2.3%) |  |  | 10.0 / 318.0 (3.1%) | 5.0 / 167.0 (3.0%) | 5.0 / 151.0 (3.3%) |  |
| Psoriasis |  | 32.0 / 118.0 (27.1%) | | 22.0 / 75.0 (29.3%) | 10.0 / 43.0 (23.3%) |  |  | 86.0 / 318.0 (27.0%) | 42.0 / 167.0 (25.1%) | 44.0 / 151.0 (29.1%) |  |
| others |  | 34.0 / 118.0 (28.8%) | | 21.0 / 75.0 (28.0%) | 13.0 / 43.0 (30.2%) |  |  | 100.0 / 318.0 (31.4%) | 49.0 / 167.0 (29.3%) | 51.0 / 151.0 (33.8%) |  |
| Missing |  | 376 | | 220 | 156 |  |  | 983 | 515 | 468 |  |
| **IBD^3^** | 489 / 494 |  | |  |  | 0.24 | 1,284 / 1,301 |  |  |  | 0.80 |
| no |  | 468.0 / 489.0 (95.7%) | | 283.0 / 293.0 (96.6%) | 185.0 / 196.0 (94.4%) |  |  | 1,202.0 / 1,284.0 (93.6%) | 628.0 / 672.0 (93.5%) | 574.0 / 612.0 (93.8%) |  |
| yes |  | 21.0 / 489.0 (4.3%) | | 10.0 / 293.0 (3.4%) | 11.0 / 196.0 (5.6%) |  |  | 82.0 / 1,284.0 (6.4%) | 44.0 / 672.0 (6.5%) | 38.0 / 612.0 (6.2%) |  |
| Missing |  | 5 | | 2 | 3 |  |  | 17 | 10 | 7 |  |
| **IBS^3^** | 488 / 494 |  | |  |  | 0.74 | 1,274 / 1,301 |  |  |  | 0.16 |
| no |  | 465.0 / 488.0 (95.3%) | | 279.0 / 292.0 (95.5%) | 186.0 / 196.0 (94.9%) |  |  | 1,243.0 / 1,274.0 (97.6%) | 643.0 / 663.0 (97.0%) | 600.0 / 611.0 (98.2%) |  |
| yes |  | 23.0 / 488.0 (4.7%) | | 13.0 / 292.0 (4.5%) | 10.0 / 196.0 (5.1%) |  |  | 31.0 / 1,274.0 (2.4%) | 20.0 / 663.0 (3.0%) | 11.0 / 611.0 (1.8%) |  |
| Missing |  | 6 | | 3 | 3 |  |  | 27 | 19 | 8 |  |
| **Cancer^3^** | 486 / 494 |  | |  |  | 0.038 | 1,282 / 1,301 |  |  |  | 0.005 |
| no |  | 442.0 / 486.0 (90.9%) | | 272.0 / 292.0 (93.2%) | 170.0 / 194.0 (87.6%) |  |  | 1,162.0 / 1,282.0 (90.6%) | 622.0 / 670.0 (92.8%) | 540.0 / 612.0 (88.2%) |  |
| yes |  | 44.0 / 486.0 (9.1%) | | 20.0 / 292.0 (6.8%) | 24.0 / 194.0 (12.4%) |  |  | 120.0 / 1,282.0 (9.4%) | 48.0 / 670.0 (7.2%) | 72.0 / 612.0 (11.8%) |  |
| Missing |  | 8 | | 3 | 5 |  |  | 19 | 12 | 7 |  |
| **Periodontits^3^** | 478 / 494 |  | |  |  | 0.44 | 1,287 / 1,301 |  |  |  | 0.051 |
| no |  | 352.0 / 478.0 (73.6%) | | 207.0 / 286.0 (72.4%) | 145.0 / 192.0 (75.5%) |  |  | 996.0 / 1,287.0 (77.4%) | 537.0 / 675.0 (79.6%) | 459.0 / 612.0 (75.0%) |  |
| yes |  | 126.0 / 478.0 (26.4%) | | 79.0 / 286.0 (27.6%) | 47.0 / 192.0 (24.5%) |  |  | 291.0 / 1,287.0 (22.6%) | 138.0 / 675.0 (20.4%) | 153.0 / 612.0 (25.0%) |  |
| Missing |  | 16 | | 9 | 7 |  |  | 14 | 7 | 7 |  |
| **Rheumatoid arthritis^3^** | 475 / 494 |  | |  |  | 0.89 | 1,260 / 1,301 |  |  |  | 0.68 |
| no |  | 434.0 / 475.0 (91.4%) | | 259.0 / 283.0 (91.5%) | 175.0 / 192.0 (91.1%) |  |  | 1,159.0 / 1,260.0 (92.0%) | 610.0 / 661.0 (92.3%) | 549.0 / 599.0 (91.7%) |  |
| yes |  | 41.0 / 475.0 (8.6%) | | 24.0 / 283.0 (8.5%) | 17.0 / 192.0 (8.9%) |  |  | 101.0 / 1,260.0 (8.0%) | 51.0 / 661.0 (7.7%) | 50.0 / 599.0 (8.3%) |  |
| Missing |  | 19 | | 12 | 7 |  |  | 41 | 21 | 20 |  |
| **Regular use of medication^6^** | 488 / 494 |  | |  |  | 0.16 | 1,290 / 1,301 |  |  |  | 0.26 |
| no |  | 68.0 / 488.0 (13.9%) | | 46.0 / 292.0 (15.8%) | 22.0 / 196.0 (11.2%) |  |  | 487.0 / 1,290.0 (37.8%) | 265.0 / 676.0 (39.2%) | 222.0 / 614.0 (36.2%) |  |
| yes |  | 420.0 / 488.0 (86.1%) | | 246.0 / 292.0 (84.2%) | 174.0 / 196.0 (88.8%) |  |  | 803.0 / 1,290.0 (62.2%) | 411.0 / 676.0 (60.8%) | 392.0 / 614.0 (63.8%) |  |
| Missing |  | 6 | | 3 | 3 |  |  | 11 | 6 | 5 |  |
| ^1^Median (IQR) and Frequencies (N/%) | | | | | | | | | | | |
| ^2^Pearson's Chi-squared test; Wilcoxon rank sum test; Fisher's exact test  ^3^Self-reported  ^4^A fasting blood sugar level from 100 to 125 mg/dL (5.6 to 7.0 mmol/L) is considered as prediabetes.  ^5^Self-reported diabetes mellitus type 2 and diagnosed by high basal glucose levels (a fasting blood sugar level of 126 mg/dL (7.0 mmol/L) or higher indicates diabetes mellitus type 2 diabetes).  ^6^Self-reported and prescription list of the general practitioner  *N= 818 Responder (two cases for baseline characteristics were excluded due to missing age) | | | | | | | | | | | |
| Abbreviations: MIG= metabolic inflammation group, ROG= registration office group, BMI= body mass index (BMI class: UW= underweight, NW= normal weight, OW= overweight, OBI= obesity grade I, OBII= obesity grade II, OBIII= obesity grade III), BP= blood pressure, HDL= high density lipoprotein, LDL= low density lipoprotein, CRP= C-reactive protein, IL-6= interleukin 6, IBD= inflammatory bowel disease and IBS= irritable bowel syndrome. | | | | | | | | | | | |
