# Supplementary material for: Cohort profile: the Food Chain Plus (FoCus) cohort
Source: Eur J Epidemiol. 2022 Oct 16;37(10):1087–105. doi: 10.1007/s10654-022-00924-y (PMC9630232; doi:10.1007/s10654-022-00924-y)
Supplement: Supplementary file 4 — Supplementary file4 (DOCX 60 kb) [file 10654_2022_924_MOESM4_ESM.docx]

**Table S4** Overview of national and international consortia as well as specific research topics using FoCus cohort data.

| **National and international Consortia** | | | |
| --- | --- | --- | --- |
| *First author* | *Year* | *Disease/Topic* | *Reference* |
| Reitmeier, Sandra | 2020 | Type 2 Diabetes mellitus, microbiome | Cell & Host [1] |
| Arloth, Janine | 2020 | Multiple sclerosis, Genome-wide association studies | PLoS Comput Biol.[2] |
| Van Dam, Esther | 2020 | Diabetes | Cell Metab.[3] |
| Pryor, Rosina | 2019 | Type 2 Diabetes mellitus, microbiome | Cell [4] |
| Thingholm, Luise | 2019 | Type 2 Diabetes mellitus, microbiome | Cell Host & Microbe [5] |
| Rhodes, Christopher J. | 2019 | Pulmonary arterial hypertension, Genome-wide association studies | Lancet Respir Med. [6] |
| Kular, Lara | 2018 | Multiple sclerosis | Nat Commun. [7] |
| Wang, Jun | 2016 | Vitamin D receptor, Genome-wide association studies | Nature Genetics [8] |
| Andlauer, Till F M | 2016 | Multiple sclerosis, Epigenetic | Sci Adv. [9] |
| Ji, Son-Gou | 2017 | Inflammatory bowel disease, Genome-wide association studies | Nature Genetics [10] |
| **Special mechanistic studies** | | | |
| *First author* | *Year* | *Disease/Topic* | *Reference* |
| Henneke, Lea | 2022 | Obesity, Type 2 Diabetes, Diet-Gut-Host Metabolism Axis | Gut Microbes [11] |
| Fangmann, Daniela | 2021 | Obesity, Type 2 Diabetes, FGF-21 | Clinical Nutrition [12] |
| Barbaresko, Janett | 2020 | Dietary pattern, Inflammatory biomarker | Eur J Nutr [13] |
| Schulz, Juliane | 2019 | Periodontitis, sFRP5 | J Clin Periodontol [14] |
| Relling, Isabelle | 2018 | Inflammation, wnt5a | J Clin Endocrinol Metab. [15] |
| Schulte, Dominik M | 2018 | Chronic inflammatory disease, Small dense LDL | Nutr Metab Cardiovasc Dis. [16] |
| Fangmann, Daniela | 2018 | Insulin sensitivity, Targeted microbiome intervention | Diabetes Care [17] |
| Kreutzer Carina | 2017 | Obesity, Hypothalamic inflammation | Diabetes [18] |
| Müller, Nike | 2015 | Inflammation, Monoclonal antibodies | J Lipid Res. [19] |

**References**

1. Reitmeier S, Kiessling S, Clavel T, et al. Arrhythmic Gut Microbiome Signatures Predict Risk of Type 2 Diabetes. Cell Host Microbe. 2020;28(2):258-72 e6. doi:10.1016/j.chom.2020.06.004

2. Arloth J, Eraslan G, Andlauer TFM, et al. DeepWAS: Multivariate genotype-phenotype associations by directly integrating regulatory information using deep learning. PLoS Comput Biol. 2020;16(2):e1007616. doi:10.1371/journal.pcbi.1007616

3. van Dam E, van Leeuwen LAG, Dos Santos E, et al. Sugar-Induced Obesity and Insulin Resistance Are Uncoupled from Shortened Survival in Drosophila. Cell Metab. 2020;31(4):710-25.e7. doi:10.1016/j.cmet.2020.02.016

4. Pryor R, Norvaisas P, Marinos G, et al. Host-Microbe-Drug-Nutrient Screen Identifies Bacterial Effectors of Metformin Therapy. Cell. 2019;178(6):1299-312.e29. doi:10.1016/j.cell.2019.08.003

5. Thingholm LB, Ruhlemann MC, Koch M, et al. Obese Individuals with and without Type 2 Diabetes Show Different Gut Microbial Functional Capacity and Composition. Cell Host Microbe. 2019;26(2):252-64 e10. doi:10.1016/j.chom.2019.07.004

6. Rhodes CJ, Batai K, Bleda M, et al. Genetic determinants of risk in pulmonary arterial hypertension: international genome-wide association studies and meta-analysis. Lancet Respir Med. 2019;7(3):227-38. doi:10.1016/S2213-2600(18)30409-0

7. Kular L, Liu Y, Ruhrmann S, et al. DNA methylation as a mediator of HLA-DRB1*15:01 and a protective variant in multiple sclerosis. Nat Commun. 2018;9(1):2397. doi:10.1038/s41467-018-04732-5

8. Wang J, Thingholm LB, Skiecevičienė J, et al. Genome-wide association analysis identifies variation in vitamin D receptor and other host factors influencing the gut microbiota. Nat Genet. 2016;48(11):1396-406. doi:10.1038/ng.3695

9. Andlauer TF, Buck D, Antony G, et al. Novel multiple sclerosis susceptibility loci implicated in epigenetic regulation. Sci Adv. 2016;2(6):e1501678. doi:10.1126/sciadv.1501678

10. Ji S-G, Juran BD, Mucha S, et al. Genome-wide association study of primary sclerosing cholangitis identifies new risk loci and quantifies the genetic relationship with inflammatory bowel disease. Nature Genetics. 2017;49(2):269-73. doi:10.1038/ng.3745

11. Henneke L, Schlicht K, Andreani NA, et al. A dietary carbohydrate - gut Parasutterella - human fatty acid biosynthesis metabolic axis in obesity and type 2 diabetes. Gut Microbes. 2022;14(1):2057778. doi:10.1080/19490976.2022.2057778

12. Fangmann D, Geisler C, Schlicht K, et al. Differential effects of protein intake versus intake of a defined oligopeptide on FGF-21 in obese human subjects in vivo. Clin Nutr. 2021;40(2):600-7. doi:10.1016/j.clnu.2020.06.006

13. Barbaresko J, Rienks J, Oluwagbemigun K, et al. Dietary patterns associated with inflammatory biomarkers in a Northern German population. Eur J Nutr. 2020;59(4):1433-41. doi:10.1007/s00394-019-02000-w

14. Schulz J, Knappe C, Graetz C, et al. Secreted frizzled-related protein 5 serum levels in human periodontitis—A nested case–control study. Journal of Clinical Periodontology. 2019;46(5):522-8. doi:10.1111/jcpe.13087

15. Relling I, Akcay G, Fangmann D, et al. Role of wnt5a in Metabolic Inflammation in Humans. J Clin Endocrinol Metab. 2018;103(11):4253-64. doi:10.1210/jc.2018-01007

16. Schulte DM, Paulsen K, Türk K, et al. Small dense LDL cholesterol in human subjects with different chronic inflammatory diseases. Nutr Metab Cardiovasc Dis. 2018;28(11):1100-5. doi:10.1016/j.numecd.2018.06.022

17. Fangmann D, Theismann E-M, Türk K, et al. Targeted Microbiome Intervention by Microencapsulated Delayed-Release Niacin Beneficially Affects Insulin Sensitivity in Humans. Diabetes Care. 2018;41(3):398-405. doi:10.2337/dc17-1967

18. Kreutzer C, Peters S, Schulte DM, et al. Hypothalamic Inflammation in Human Obesity Is Mediated by Environmental and Genetic Factors. Diabetes. 2017;66(9):2407-15. doi:10.2337/db17-0067

19. Müller N, Schulte DM, Türk K, et al. IL-6 blockade by monoclonal antibodies inhibits apolipoprotein (a) expression and lipoprotein (a) synthesis in humans. J Lipid Res. 2015;56(5):1034-42. doi:10.1194/jlr.P052209
